# Digital assessment of real-world walking in people with impaired mobility: How many hours and days are needed?

**DOI:** 10.1186/s12966-025-01851-3

**Published:** 2025-11-21

**Authors:** Joren Buekers, Julia Chernova, Sarah Koch, José Marchena, Jorge Lemos, Clemens Becker, Tecla Bonci, Julia Braun, Gavin Brittain, Ellen Buckley, Brian Caulfield, Silvia Del Din, Heleen Demeyer, Anja Frei, Eran Gazit, Jeffrey M. Hausdorff, Anisoara Ionescu, Carl-Philipp Jansen, Jochen Klenk, Michael Long, Basil Sharrack, David Singleton, Thierry Troosters, Alison J. Yarnall, Lynn Rochester, Judith Garcia-Aymerich

**Affiliations:** 1 Barcelona Institute for Global Health (ISGlobal), Barcelona, Spain; 2 Universitat Pompeu Fabra (UPF), Barcelona, Spain; 3 CIBER Epidemiología y Salud Pública (CIBERESP), Barcelona, Spain; 4 Bayer plc, Reading, UK; 5 Department of Sport, Exercise and Health, University of Basel, Basel, Switzerland; 6 Robert Bosch Gesellschaft für Medizinische Forschung, Stuttgart, Germany; 7 School of Mechanical, Aerospace and Civil Engineering & INSIGNEO Institute for in silico Medicine, The University of Sheffield, Sheffield, UK; 8 Epidemiology, Biostatistics and Prevention Institute (EBPI), University of Zurich (UZH), Hirschengraben 84, Zurich, 8001 Switzerland; 9 NIHR Sheffield Biomedical Research Centre, Sheffield Teaching Hospitals NHS Foundation Trust, Sheffield, UK; 10 Department of Neurology, Neuroscience Institute, Sheffield Teaching Hospitals NHS Foundation Trust, University of Sheffield, Sheffield, UK; 11 School of Medicine & Population Health & INSIGNEO Institute for in silico Medicine, University of Sheffield, Sheffield, UK; 12 Insight Centre for Data Analytics, University College Dublin, Dublin, Ireland; 13 School of Public Health, Physiotherapy and Sports Science, University College Dublin, Dublin, Ireland; 14 Translational and Clinical Research Institute, Faculty of Medical Sciences, Newcastle University, Newcastle upon Tyne, UK; 15 National Institute for Health and Care Research (NIHR) Newcastle Biomedical Research Centre (BRC), Newcastle University, Newcastle upon Tyne, UK; 16 Department of Rehabilitation Sciences & Pulmonary Rehabilitation, Respiratory Division, KU Leuven, University Hospital Gasthuisberg, Leuven, Belgium; 17 Department of Rehabilitation Sciences, Ghent University, Ghent, Belgium; 18 Center for the Study of Movement, Cognition and Mobility, Neurological Institute, Tel Aviv Sourasky Medical Center, Tel Aviv, Israel; 19 Sagol School of Neuroscience, Department of Physical Therapy, Faculty of Medical & Health Sciences, Tel Aviv University, Tel Aviv, Israel; 20 Rush Alzheimer’s Disease Center, Department of Orthopedic Surgery, Rush University Medical Center, Chicago, IL USA; 21 Laboratory of Movement Analysis and Measurement, Ecole Polytechnique Federale de Lausanne, Lausanne, Switzerland; 22 Geriatric Center, Heidelberg University Clinic, Heidelberg, Germany; 23 Institute of Epidemiology and Medical Biometry, Ulm University, Ulm, Germany; 24 Department of Computer Science, The University of Sheffield, Sheffield, UK; 25 The Newcastle Upon Tyne Hospitals NHS Foundation Trust, Newcastle upon Tyne, UK

**Keywords:** Gait, Walking activity, Reliability, Wearable sensors, Digital health

## Abstract

**Background:**

Impaired mobility increases falls and mortality risk. However, guidelines to reliably assess real-world walking activity and gait remain undefined. We aimed to (i) determine the minimum daily wear time during waking hours (7:00–22:00) for a valid measurement day, (ii) identify the minimum number of valid days, and (iii) weekend days, to reliably assess weekly walking activity and gait parameters, and (iv) provide recommendations for reliable real-world walking assessments.

**Methods:**

Participants with chronic obstructive pulmonary disease (*n* = 565), multiple sclerosis (*n* = 558), Parkinson’s disease (*n* = 543) or proximal femoral fracture (*n* = 487) from 10 countries were asked to wear a single wearable device on the lower back, 24 h/day for seven days, resulting in 13,191 measurement days. The Mobilise-D processing pipeline was used to obtain 24 daily walking activity and gait parameters. Minimum daily wear time was determined as the highest wear time category that did not statistically change parameter values. Intraclass correlation coefficients ≥ 0.80 determined the minimum number of valid measurement and weekend days.

**Results:**

The minimum daily wear time varied between “no requirement” (13% of parameter-condition combinations) and > 14 h (19%), with higher requirements for walking activity than gait parameters. The minimum number of days ranged from 1 (17%) to > 7 days (6%), and was higher for parameters that are yet to be clinically validated. There was no evidence of a weekend nor health condition effect on parameter reliability.

**Conclusions:**

For studies involving multiple walking activity and gait parameters or health conditions, expert consensus recommends a minimum of > 12 h of daily wear time across ≥ 3 days. For studies that involve specific parameters or health conditions, individual recommendations are provided within the manuscript.

**Supplementary Information:**

The online version contains supplementary material available at 10.1186/s12966-025-01851-3.

## Background

Walking is a meaningful aspect of health for people with diverse health conditions [1] and can be described in terms of walking activity (how much one walks) and gait (how one walks). Walking speed and gait are independent predictors of various health outcomes: walking activity is a well-known health determinant, protecting against obesity, depression, cardiovascular diseases, and mortality [2–5]; and gait, particularly slow walking speed, predicts falls, disability and mortality in older adults [6–8]. As older adults are more prone to have reduced walking activity and altered gait, the global burden of impaired mobility will rise with our rapidly ageing populations [9, 10]. Therefore, developing and validating tools to measure real-world walking activity and gait is essential.

Traditionally, most walking assessments were either confined to laboratory settings [11], which do not reflect real-world circumstances [12–16], or primarily focussed on the amount, duration or intensity of real-world walking activity, based on parameters such as daily step count [4, 5]. Other relevant walking activity parameters like the number of walking bouts (WBs) or their average duration [17–20], are infrequently measured, and gait parameters remain largely unexplored in real-world contexts. Moreover, studies on (real-world) walking are often restricted to one specific population. The recently developed Mobilise-D processing pipeline generates a comprehensive list of walking activity and gait parameters using a single wearable device worn at the lower back [21–24]. These parameters, or Digital Mobility Outcomes (DMOs), have been validated against gold standards in both laboratory and free-living settings for various health conditions, representative of impaired mobility of diverse origin: respiratory pathology (chronic obstructive pulmonary disease, COPD), cardiac pathology (congestive heart failure), healthy older adults, neuroinflammatory problems (multiple sclerosis, MS), neurodegenerative conditions (Parkinson’s disease, PD) and osteoporosis and sarcopenia (proximal femoral fracture, PFF) [22, 23, 25]. However, guidelines for establishing a valid measurement day and for providing a reliable digital assessment of walking activity and gait during longer periods of real-world walking are still needed.

Therefore, this study aimed to (1) identify the minimum required daily wear time during waking hours (7:00–22:00) that constitutes a valid measurement day, (2) identify the minimum number of valid measurement days and (3) the minimum number of valid weekend days that are required to obtain reliable weekly walking activity and gait DMOs, and (4) propose recommendations for the assessment of walking activity and gait DMOs in studies involving people with impaired mobility.

## Methods

### Study design and participants

This multicentre, cross-sectional study used data from the baseline visit of the clinical validation study in the IMI2-JU-funded Mobilise-D project [26], approved by all relevant Ethical Committees (Appendix 1). All participants provided written informed consent.

People with COPD, MS, PD and PFF were recruited across 17 sites in 10 countries (Appendix 1). Inclusion criteria included: ability to walk 4 m independently, anticipated availability, ability to consent and comply with study procedures, willingness to wear a wearable sensor, and ability to read and write in the first language of the respective country [26].

**Fig. 1:**
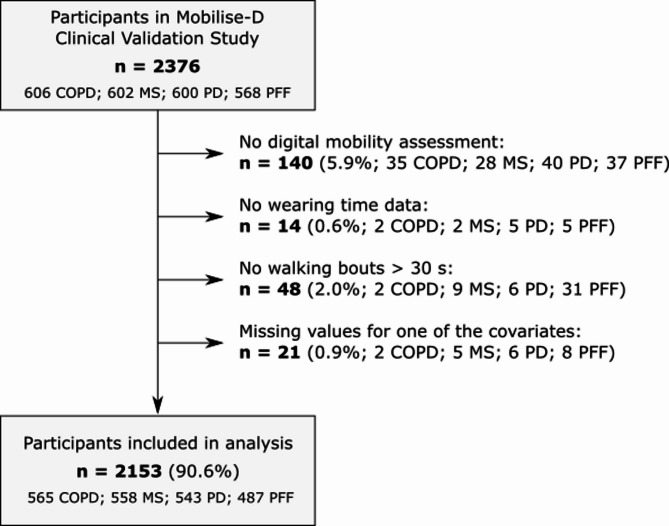
Flow diagram of participants that were included in the analyses, *n* (%). COPD = chronic obstructive pulmonary disease; MS = multiple sclerosis; PD = Parkinson’s disease; PFF = proximal femoral fracture

From the original sample of 2376 participants (Fig. 1), we excluded 137 (5.8%) without a digital mobility assessment, 14 (0.6%) without wear time information, 86 (3.6%) without WBs > 30 s, and 25 (1.1%) with missing covariate values (Appendix 2), resulting in 2114 (89.0%) participants for the current analysis (565 COPD, 558 MS, 543 PD, and 487 PFF).

### Measurements

We collected data on age, gender, height, weight, education level, employment status, living arrangement, number of prescribed medications, use of any type of mobility aids indoor and outdoor (including ankle foot orthosis or functional electrical stimulation device), and self-reported functional status (Functional Component of the Late Life Function and Disability Instrument; LLFDI-FC [27]). Health condition severity was assessed through forced expiratory volume in the first second (FEV_1_) [28] for COPD, Expanded Disability Status Scale (EDSS) [29] for MS, Movement Disorder Society – Unified Parkinson’s Disease Rating Scale Part III (MDS-UPDRS III) [30] for PD, and Short Physical Performance Battery (SPPB) [31] score for PFF.

Participants were asked to wear a single wearable device on the lower back, 24 h/day for seven days: MoveMonitor+ (McRoberts B.V., Den Haag, the Netherlands) or AX6 (Axivity Ltd, Newcastle Upon Tyne, UK). For each day, we calculated 24 DMOs using all data from the full calendar day via the Mobilise-D processing pipeline [22–24], covering five walking domains: walking activity amount (three DMOs, e.g., WB step count [steps/day]), walking activity pattern (seven DMOs, e.g., number of WBs [WBs/day]), gait-pace (six DMOs, e.g., walking speed in shorter (10–30 s) WBs [m/s]), gait-rhythm (five DMOs, e.g., cadence in all WBs [steps/min]) and bout to bout gait variability (four DMOs, e.g., walking speed bout to bout variability between longer (>30 s) WBs [%]) (full list and definitions in Appendix Table 4). The Mobilise-D processing pipeline uses condition-specific algorithms to account for differences between conditions in movement patterns and walking speed, among others [22, 23]. Daily wear time during waking hours (defined as 07:00–22:00, local time, from published recommendations [32]; which includes 92% of the walking duration performed by the participants) was calculated by summing all minutes that the device was worn, as estimated by the McRoberts wear time algorithm [33], and divided into the following categories: <8 h, 8–10 h, 10–11 h, 11–12 h, 12–13 h, 13–14 h, and ≥ 14 h (reference category).

### Statistical analyses

Detailed analyses and R code are provided in Appendix 2. Briefly, objectives 1–3 were addressed sequentially, and each DMO was separately analysed for each health condition (resulting in 24 × 4 = 96 analysed DMO-condition combinations), as outlined below. The analyses for objective 1 assessed potential systematic measurement error of daily DMO values due to insufficient wear time, while objective 2–3 assessed potential random measurement error of weekly DMO values due to an insufficient number of valid measurement days (objective 2) or weekend days (objective 3).

As future studies will simultaneously examine multiple DMOs in multiple conditions [21], a separate processing pipeline for each of the 96 DMO-condition combinations in these studies is deemed impractical. Therefore, the results of all 96 DMO-condition combinations for objectives 1–3 were assessed by an expert group in order to propose DMO- and condition-agnostic recommendations that can be used by studies involving multiple walking activity and gait parameters or health conditions (objective 4). These recommendations seek to balance methodological rigour with analytical feasibility, and were based on the results of the 96 DMO-condition combinations, sample size, potential selection bias, random and systematic measurement errors, and clinical relevance. The expert group consisted of a multidisciplinary collaboration between clinical experts of the four conditions, experts on physical activity and gait, epidemiologists, statisticians, and engineers, making sure to account for all relevant technical, clinical and statistical considerations.

To determine the minimum wear time that constitutes a valid day (objective 1), we built linear mixed models with each DMO as outcome, a random intercept for participants, wear time during waking hours (categorical) as exposure, and adjusted for confounders. The minimum required daily wear time that constitutes a valid measurement day was determined for each DMO-condition combination as the upper limit of the highest wear time category with statistically significant differences in DMO values compared to the reference category.

To obtain the minimum number of days to obtain reliable weekly values (objective 2), we used only measurement days that were considered valid based on the outcome of objective 1. We built unadjusted linear mixed models with DMOs as outcome, and a random intercept for participants. Single-day within person variance and between person variance were directly extracted from the mixed effects regression models. Intraclass correlation coefficients (ICC) values for different numbers of measurement days (k) were then calculated, based on the ICC(k) formula from McGraw and Wong [34, 35]. We identified the lowest number of days resulting in an intraclass correlation coefficient (ICC) ≥ 0.80 [36, 37] for each DMO. This analysis was repeated after stratification by physical capacity (above or below condition-specific median SPPB score) as a post-hoc analysis.

To determine the minimum number of weekend days to obtain reliable weekly values (objective 3), we used only participants with the minimum number of valid days determined in objectives 1 and 2. We built unadjusted linear mixed models with each DMO as the outcome, a random intercept for participants, and day type (week or weekend day) as exposure. Single-day within person variance for weekdays, single-day within person variance for weekend days and between person variance were directly extracted from the mixed effects regression models. Hereafter, ICC values for different numbers of weekdays and weekend days were calculated (see Appendix 2), but only for combinations for which the sum of the number of week and weekend days equals the minimum required number of days determined by expert consensus in objective 2. For each DMO-condition combination, weekend days were only required if their inclusion resulted in an ICC ≥ 0.80, and excluding them resulted in an ICC < 0.80. This analysis was repeated after stratification by physical capacity (above or below condition-specific median SPPB score) as a post-hoc analysis.

Statistical analyses were performed using a complete-case approach in R 4.3.2 (lme4 and nlme packages), using the Mobilise-D clinical validation study dataset version 5.1.

## Results

### Participant and measurement characteristics

There were no statistically significant differences in age, gender, height, or BMI between the 2153 included and 223 excluded participants. Compared to those excluded, included participants had a lower FEV_1_% predicted (COPD), lower indoor use of mobility aids, higher LLFDI-FC and lower EDSS score (MS), and higher SPPB total scores (MS, PD, PFF; Appendix Table 5).

Mean (SD) age, gender distribution and condition severity were: for COPD, 68 (8) years, 36% female and FEV_1_ of 54 (20) % predicted; for MS, 52 (11) years, 64% female and median (P25-P75) EDSS score of 5 [4–6]; for PD, 66 (10) years, 36% female and MDS-UPDRS III score of 26.4 (12.5); and for PFF, 77 (10) years; 66% female and SPPB score of 6.4 (3.1) (Table 1).

**Table 1:** Participant and measurement characteristics (“*n*” refers to the number of participants)

	*All (n = 2153)*	*COPD (n = 565)*	*MS (n = 558)*	*PD (n = 543)*	*PFF (n = 487)*
Age (years), mean (SD)	65 (13)	68 (8)	52 (11)	66 (10)	77 (10)
Gender: Female, *n* (%)	1080 (50)	206 (36)	358 (64)	197 (36)	319 (66)
Height (cm), mean (SD)	170 (10)	168 (9)	170 (9)	172 (10)	168 (10)
Body mass index (kg/m^2^), mean (SD)	26 (4)	27 (5)	26 (6)	26 (4)	24 (4)
Years of education, median (P25-P75)	14 (11–17)	13 (11–15)	15 (12–18)	16 (13–18)	13 (10–15)
Employment status					
Active worker (full/part time), *n* (%)	604 (28)	92 (16)	275 (49)	183 (34)	54 (11)
Retired, *n* (%)	1288 (60)	390 (69)	175 (31)	326 (60)	397 (82)
Other (e.g., unemployed, sick leave, home-maker)	261 (12)	83 (15)	108 (19)	34 (6)	36 (7)
Living arrangement					
Alone, *n* (%)	572 (27)	161 (28)	89 (16)	89 (16)	233 (48)
With somebody, *n* (%)	1581 (73)	404 (72)	469 (84)	454 (84)	254 (52)
Number of prescribed medications, median (P25-P75)	6 (3–8)	7 (4–9)	4 (2–6)	5 (3–7)	7 (4–10)
Use of mobility aids indoors, *n* (%)	427 (20)	12 (2)	134 (24)	19 (3)	262 (54)
Use of mobility aids outdoors, *n* (%)	759 (35)	49 (9)	327 (59)	56 (10)	327 (67)
LLFDI-FC, mean (SD)	57 (12)	59 (9)	53 (12)	63 (12)	53 (13)
FEV_1_ (% predicted), mean (SD)		54 (20)			
EDSS score, median (P25-P75)			5 (4–6)		
MDS-UPDRS III, mean (SD)				26.4 (12.5)	
SPPB (total score), mean (SD)	8.5 (2.8)	9.9 (1.8)	7.7 (2.9)	9.6 (1.7)	6.4 (3.1)
Device type:					
McRoberts MoveMonitor+, *n* (%)	1062 (49)	468 (83)	96 (17)	386 (71)	112 (23)
Axivity AX6, *n* (%)	1091 (51)	97 (17)	462 (83)	157 (29)	375 (77)
Measurement days (days), median (P5-P95)	7 (6–7)	7 (6–7)	7 (6–7)	7 (6–7)	7 (6–7)

*COPD* chronic obstructive pulmonary disease, *MS* multiple sclerosis, *PD* Parkinson’s disease, *PFF* proximal femoral fracture, *LLFDI-FC* Functional Component of the Late Life Function and Disability Instrument, *FEV*_1_ forced expiratory volume in the first second, *EDSS* Expanded Disability Status Scale, *MDS-UPDRS* Movement Disorder Society – Unified Parkinson’s Disease Rating Scale; *SPPB* Short Physical Performance Battery

Compliance was very high, with the wearable device worn for a median (P5-P95) of 24 (17–24) hours/day (range: 2 minutes–24 h), and 15 (13-15) hours/day during waking hours (range: 0–15 h), for a median (P5-P95) of 7 (6-7) days (range: 1–7 days; Tables 1 and 2). Participants with lower compliance generally had worse functional status, while differences in age, gender, height, BMI, education level, mobility aid use, health condition severity, and device type were inconsistent across conditions (Appendix Table 6).

**Table 2:** Measurement characteristics at the day level (“*n*” refers to the number of days)

	*All (n = 13191)*	*COPD (n = 3651)*	*MS (n = 3421)*	*PD (n = 3466)*	*PFF (n = 2561)*
Wear time during waking hours (h/day), median (P5-P95)	15 (13–15)	15 (13–15)	15 (13–15)	15 (12–15)	15 (15-15)
Wear time during waking hours using:					
McRoberts MoveMonitor+ (h/day), median (P5-P95)	15 (11–15)	15(11–15)	15 (11–15)	15 (11–15)	15 (11–15)
Axivity AX6 (h/day), median (P5-P95)	15 (14–15)	15 (14–15)	15 (14–15)	15 (14–15)	15 (14–15)
Wear time during waking hours					
<8h, *n* (%)	197 (1)	49 (1)	44 (1)	57 (2)	47 (2)
8h-10h, *n* (%)	122 (1)	30 (1)	25 (1)	49 (1)	18 (1)
10-11h, *n* (%)	88 (1)	26 (1)	15 (0)	34 (1)	13 (1)
11-12h, *n* (%)	142 (1)	45 (1)	17 (0)	61 (2)	19 (1)
12-13h, *n* (%)	217 (2)	76 (1)	45 (1)	63 (2)	33 (1)
13-14h, *n* (%)	630 (5)	211 (6)	137 (4)	194 (5)	88 (3)
≥14h, *n* (%)	11795 (89)	3214 (88)	3138 (92)	3100 (87)	2343 (91)
Day type					
Weekday, *n* (%)	9540 (72)	2629 (72)	2454 (72)	2502 (72)	1901 (74)
Weekend day, *n* (%)	3651 (28)	1022 (28)	967 (28)	964 (28)	660 (26)

DMO values ranged between 39 and 38,858 steps/day for WB step count, 2–1553 WBs/day for number of WBs, 0.3–1.3 m/s for walking speed in shorter (10–30 s) WBs, 61.3–111.3.3.3 steps/min for cadence in all WBs, and 0–76% for walking speed bout to bout variability between longer (>30 s) WBs. See ranges for all DMOs in Appendix Table 7.

### Minimum daily wear time

We analysed 13,191 days from 2153 participants. Table 3 presents the minimum daily wear time requirements for each DMO-condition combination. Without any minimum wear time, 13% of DMO-condition combinations yielded valid daily-level DMO values. This increased to 32%, 44%, 57%, 65%, 81% and 100% with minimum wear time requirements of > 8 h, > 10 h, > 11, >12, > 13 and > 14 h, respectively.

**Table 3:** Minimum required daily wear time (in hours) during waking hours (07:00–22:00) for the 96 digital mobility outcome (DMO)-condition combinations. “None” indicates that there is no minimum requirement. * indicates domains that are yet to be clinically validated

	*COPD* *(n = 565)*	*MS* *(n = 558)*	*PD* *(n = 543)*	*PFF* *(n = 487)*
Walking activity - Amount				
Walking duration	>13	>14	>13	>14
WB step count	>13	>14	>13	>14
Walking activity – Pattern*				
Number of WBs	>13	>14	>13	>10
Number of WBs >10s	>13	>14	>13	>10
Number of WBs >30s	>12	>14	>13	>14
Number of WBs >60s	>13	>14	>13	>14
WB duration	None	>10	>11	>8
P90 WB duration	None	>8	>14	None
WB duration bout to bout variability	None	None	>8	>14
Gait – Pace				
Walking speed in shorter (10-30s) WBs	>11	>10	>11	>11
Walking speed in longer (>30s) WBs	>8	None	>12	>8
P90 walking speed in WBs >10 s	>8	>10	>12	>12
P90 walking speed in longer (>30s) WBs	>13	>14	>12	>8
Stride length in shorter (10-30s) WBs	>11	>14	>13	>14
Stride length in longer (>30s) WBs	>13	>8	>12	>10
Gait – Rhythm				
Cadence in all WBs	None	>14	>11	None
Cadence in longer (>30s) WBs	>8	>14	>8	>10
P90 cadence in longer (>30s) WBs	>12	>14	>8	>10
Stride duration in all WBs	>8	>10	>11	>10
Stride duration in longer (>30s) WBs	>8	None	>11	>8
Gait – Bout to bout variability*				
Walking speed bout to bout variability between longer (>30s) WBs	>10	None	>8	>11
Stride length bout to bout variability between longer (>30s) WBs	>8	>13	>11	>11
Cadence bout to bout variability	>8	None	>8	None
Stride duration bout to bout variability	>8	>11	>11	>13

*COPD* chronic obstructive pulmonary disease, *MS* multiple sclerosis, *PD* Parkinson’s disease, *PFF* proximal femoral fracture, *WB* walking bout

For walking domains, the minimum required daily wear time was generally higher for walking activity DMOs, with 67% DMO-condition combinations requiring 12 h and 33% requiring > 14 h. In contrast, for gait DMOs, only 27% required > 12 h and 10% required > 14 h. Daily wear time requirements were similar across conditions.

Expert consensus determined that a minimum daily wear time below 12 h should not be considered, as 43% of DMO-condition combinations required 12 h or more. The choice among >12, >13 or >14 h involved a trade-off between systematic measurement error (less restrictive requirements could significantly alter DMO values, as shown in Table 3), sample size (increasing the threshold from >12 h to >14 h would exclude up to 316 (15%) additional participants, Appendix Table 8), and selection bias (more restrictive requirements excluded participants with worse health status, Appendix Table 6). Since our recommendations aimed to be relevant for a broad range of health conditions with impaired mobility [21], selection bias and a reduced sample size were deemed more detrimental than systematic measurement error. Therefore, expert consensus recommended a daily wear time of >12 h.

### Minimum number of valid measurement days

Only days with > 12 h of wear time between 07:00–22:00 (local time) were used for objective 2 (12,642 days from 2127 participants). Table 4 shows the minimum number of valid measurement days required for each DMO-condition combination. One valid day sufficed for reliable weekly-level DMO values in 17% DMO-condition combinations, increasing to 53%, 67%, 77%, 83%, 89% and 94% for two, three, four, five, six or seven valid measurement days, respectively.

The minimum number of measurement days was generally higher for walking domains still needing clinical validation. Specifically, 79% of DMO-condition combinations for walking activity patterns and 81% for bout to bout gait variability required three or more days, and 46% and 56% requiring five or more days, respectively. In contrast, the well-established domains—walking activity amount, gait-pace, and gait-rhythm—required three or more days for 25%, 13% and 20% of combinations, respectively, with none of these domains requiring five or more days. The minimum number of measurement days was similar across conditions.

By expert consensus, fewer than 3 measurement days were considered insufficient, as 47% DMO-condition combinations required ≥ 3 days. The choice among ≥ 3, ≥4 or ≥ 5 days as the recommended minimal number of measurement days involved a trade-off between random measurement error (less restrictive requirements could increase random measurement error, as shown in Table 4), sample size (exclusions of 90 (4.5%) and 211 (10.6%) additional participants when raising the threshold to ≥ 4 or ≥ 5 days, respectively; Appendix Table 8) and selection bias (more stringent requirements excluded participants with a worse health condition, Appendix Table 6). As with objective 1, selection bias was deemed more detrimental than random measurement error. Moreover, the clinical relevance of most DMOs requiring a higher number of measurement days (i.e., number of WB >60 s, P90 WB duration, WB duration variability and the four bout to bout gait variability DMOs) is yet to be determined. Hence, expert consensus indicated a minimum of ≥ 3 measurement days.

**Table 4:** Minimum required number of measurement days for the 96 digital mobility outcome (DMO)-condition combinations. A forward slash (/) indicates that one week of measurements was insufficient to reach an intraclass correlation coefficient ≥ 0.80. * indicates domains that are yet to be clinically validated

	*COPD (n = 565)*	*MS (n = 558)*	*PD (n = 543)*	*PFF (n = 487)*
Walking activity - Amount				
Walking duration	≥2	≥2	≥3	≥2
WB step count	≥2	≥2	≥4	≥2
Walking activity – Pattern*				
Number of WBs	≥2	≥2	≥3	≥1
Number of WBs >10s	≥2	≥2	≥3	≥2
Number of WBs >30s	≥4	≥5	≥6	≥5
Number of WBs >60s	≥6	/	/	≥7
WB duration	≥3	≥4	≥4	≥3
P90 WB duration	≥4	≥5	≥7	≥4
WB duration bout to bout variability	≥5	≥5	/	≥5
Gait - Pace				
Walking speed in shorter (10-30s) WBs	≥1	≥1	≥2	≥1
Walking speed in longer (>30s) WBs	≥3	≥2	≥3	≥1
P90 walking speed in WBs >10 s	≥2	≥1	≥2	≥1
P90 walking speed in longer (>30s) WBs	≥2	≥2	≥3	≥1
Stride length in shorter (10-30s) WBs	≥1	≥2	≥1	≥1
Stride length in longer (>30s) WBs	≥2	≥2	≥2	≥2
Gait – Rhythm				
Cadence in all WBs	≥1	≥1	≥1	≥1
Cadence in longer (>30s) WBs	≥2	≥2	≥3	≥2
P90 cadence in longer (>30s) WBs	≥2	≥2	≥3	≥2
Stride duration in all WBs	≥1	≥2	≥2	≥2
Stride duration in longer (>30s) WBs	≥3	≥3	≥4	≥2
Gait – Bout to bout variability*				
Walking speed bout to bout variability between longer (>30s) WBs	≥6	≥6	/	≥7
Stride length bout to bout variability between longer (>30s) WBs	/	≥6	/	≥7
Cadence bout to bout variability	≥2	≥2	≥2	≥3
Stride duration bout to bout variability	≥4	≥4	≥7	≥4

*COPD* chronic obstructive pulmonary disease, *MS* multiple sclerosis, *PD* Parkinson’s disease, *PFF* proximal femoral fracture, *WB* walking bout

Stratified analyses showed that the minimal number of valid measurement days was slightly higher for people with a higher physical capacity in MS (17 out of 24 DMOs) and PD (14 out of 24 DMOs), and for people with a lower physical capacity after PFF (13 out of 24 DMOs). In COPD, people with a higher physical capacity required more measurement days for 7 DMOs, and less for 3 DMOs (Appendix Table 9). In spite of this, the number of DMOs with reliable weekly values when including at least 3 valid measurement days were comparable between participants with a lower (69% DMO-condition combinations) or higher physical capacity (63% DMO-condition combinations). Therefore, the stratified analyses confirmed that the recommended threshold of ≥ 3 valid measurement days is also appropriate for participants across different levels of physical capacity.

### Minimum number of valid weekend days

Based on results of objectives 1 and 2, only days with > 12 h of wear time were used, and combinations of valid weekdays and weekend days that totalled three were tested (three weekdays; two weekdays and one weekend day; or one weekday and two weekend days).

Twelve out of 24 DMOs achieved ICC ≥ 0.80 in all combinations for all conditions, while the remaining DMOs yielded ICC values between 0.62 and 0.85 (Appendix Table 10). No significant differences were found between combinations of weekdays and weekend days. Therefore, expert consensus proposed that there is no minimum requirement for weekend days to obtain reliable weekly walking activity and gait DMOs. Stratified analyses confirmed that no minimum number of weekend days is required for participants with a lower or higher physical capacity.

## Discussion

### Main results

Our thorough analysis of measurement characteristics affecting the reliability of walking activity and gait DMOs in a large sample of people with various health conditions shows that: (1) the minimum required daily wear time during waking hours that constitutes a valid measurement day ranged from no minimum to >14 h, typically higher for walking activity than for gait; (2) the minimum number of valid measurement days for reliable weekly DMO values varied between 1 and >7 days, generally higher for walking domains yet to be clinically validated; (3) the inclusion of weekend days did not affect DMO reliability; and (4) expert consensus advised a wear time of >12 h for ≥ 3 days to obtain reliable weekly walking activity and gait DMOs across diverse conditions.

The minimum wear time for a valid day varied across DMOs and walking domains. A higher wear time requirement for walking activity compared to gait was expected, as additive DMOs (e.g., daily step count) are more sensitive to wear time than those representing daily averages, maxima, or variability. Similarly, the minimum number of valid days also varied across DMOs and walking domains, consistent with previous research indicating that the required days depend on the physical activity parameter of interest [32, 37–40]. However, within domains, the minimum days were generally consistent and higher for domains not yet clinically validated, such as walking activity patterns and bout to bout gait variability, which may be more susceptible to behaviour and context. Importantly, neither the minimum wear time nor the minimum number of days differed consistently by health condition, facilitating condition-agnostic recommendations.

The inclusion of more or fewer weekend days did not affect the reliability of walking activity or gait DMOs. In this study, the weekend effect on DMO reliability was assessed only in terms of potential differences in variance between weekdays and weekends. Clinically relevant differences in absolute DMO values between these periods can only be determined once the minimum clinically important differences for the DMOs are established, which is a key goal of the ongoing analyses of the Mobilise-D clinical validation study data [26]. Hence, future recommendations on including weekend days may be revised for certain DMOs.

### Comparison with previous literature

Expert consensus recommended a minimum daily wear time of >12 h between 07:00–22:00 for ≥ 3 days, without restrictions on weekend days, when applying the Mobilise-D method (which is based on full calendar day measurements) across the full list of DMOs and across various health conditions. In comparison, common guidelines for conventional physical activity parameters (related to walking activity DMOs) in children, adults, or people with chronic conditions suggest less strict daily wear time requirements (>8 h or >10 h) [32, 41–44], stricter measurement day requirements (≥ 3 to ≥ 10 days) [32, 37–40, 42–47], and inconsistent recommendations regarding weekend inclusion [32, 37, 40, 42–45, 47]. Potential explanations for these differences include: (i) technical variations in devices or algorithms that may influence DMO values and variability (though we used extensively validated devices and algorithms [21–23]); (ii) the wider variability in mobility impairment in our study (given the diverse conditions included), possibly increasing the need for longer wear time; and (iii) the fact that we purposefully formulated unified recommendations for DMOs that differ inherently (i.e., walking activity vs. gait DMOs). Finally, the lack of literature on the minimum wear time for gait DMOs precludes any comparison.

### Implications

The general recommendations of > 12 h/day for ≥ 3 days will support the reliable collection of walking activity and gait DMOs, based on measurements with a single wearable device worn on the lower back that fulfils certain requirements (see below) and the Mobilise-D processing pipeline, in future studies involving multiple DMOs and/or conditions, including other populations with impaired mobility that were not currently assessed (as long as the DMO values are within the ranges of the current study). These recommendations are furthermore applicable to populations with either lower or higher physical capacities. In addition, studies focusing on specific DMOs, domains, or conditions could tailor these thresholds based on the results in Tables 3 and 4, and Appendix Table 9. For example, if only walking activity amount is considered, a threshold of > 14 h/day for ≥ 2 days may be more suitable than the broader recommendations of > 12 h/day for ≥ 3 days. Nevertheless, although only 3 valid measurement days are needed to obtain reliable weekly DMO values, future study protocols should still aim for 7 consecutive measurement days, to account for potential participant adherence issues and/or technical problems with the wearable devices.

### Strengths and limitations

This is the first study to provide guidelines for obtaining valid measurement days and reliable weekly walking activity and gait DMOs across various health conditions, based on a large dataset of 13,191 days from 2153 participants. The methodological rigour, that is, using mixed-effects regression models to define valid days (objective 1) and reliable weeks (objectives 2 and 3), minimised systematic errors by avoiding the exclusion of days or participants based on arbitrary thresholds, and reduced random errors by not relying on random subsamples, as seen in previous studies [38, 40, 44, 46–49]. Another strength over similar previous studies was the sequential approach: first defining what constitutes a valid measurement day (with unbiased DMO values), and only then assessing the reliability of weekly DMO values. This ensured that the recommended thresholds did not result in reliable but biased DMO values, which could occur if the minimal daily wear time and the minimal number of measurement days were determined simultaneously based solely on ICC values. The analyses, results, and recommendations stem from a multidisciplinary collaboration, ensuring that all relevant technical, clinical, and statistical factors were considered.

A limitation of the study was the relatively small sample size in some lower wear time categories, which may have masked potential differences in DMO values for these groups. Moreover, the clinical relevance of differences in absolute DMO values between weekdays and weekends could not yet be assessed, as these thresholds have not been established. Even though the presented results and recommendations are valid for any wearable device that is worn on the lower back and fulfilling the minimal requirements defined by the Mobilise-D consortium (i.e., triaxial accelerometer with 100 Hz sampling frequency, 8 g range and 1 mg resolution; and triaxial gyroscope with 100 Hz sampling frequency, 2000 degrees per second range and 70 milidegrees per second resolution [25]), it is unclear whether the presented results and recommendations can be extrapolated to DMOs that are measured with a device that is not worn on the lower back or does not fulfil the mentioned requirements. A final limitation to consider when applying the general recommendation (>12 h of wear time for ≥ 3 days) in future studies is that these thresholds may not be appropriate when assessing specific DMOs in specific health conditions. This manuscript provides the necessary information for researchers to adapt the thresholds to their particular research questions and populations, as outlined above in the Implications section.

## Conclusions

The minimum daily wear time and number of days required for reliable weekly walking activity and gait DMOs were DMO-specific, unaffected by weekend days, and consistent across conditions. A wear time of > 12 h for ≥ 3 days is recommended by expert consensus when using the Mobilise-D processing pipeline in studies involving multiple DMOs or health conditions. For studies that involve specific DMOs or health conditions, these thresholds can be tailored based on the results provided in Tables 3 and 4, and Appendix Table 9.

## Supplementary Information

Supplementary Material 1.

## Data Availability

Data will be made publicly available on online repositories (Zenodo) at the end of the SUSTAIN Mobilise-D project, a follow-on consortium created to maximise the impact and to advance the achievements of Mobilise-D.

## Abbreviations

COPD: chronic obstructive pulmonary disease
DMO: Digital mobility outcome
EDSS: Expanded Disability Status Scale
FEV_1_: forced expiratory volume in the first second
ICC: Intraclass correlation coefficients
LLFDI-FC: Functional Component of the Late Life Function and Disability Instrument
MDS-UPDRS: Movement Disorder Society – Unified Parkinson’s Disease Rating Scale
MS: multiple sclerosis
PD: Parkinson’s disease
PFF: proximal femoral fracture
SPPB: Short Physical Performance Battery
WB: walking bout

## References

[1] Delgado-Ortiz L, Polhemus A, Keogh A, et al. Listening to the patients’ voice: a conceptual framework of the walking experience. Age Ageing. 2023;52(1):1–10. 10.1093/ageing/afac233 PMC9894103 36729471

[2] Banach M, Lewek J, Surma S, et al. The association between daily step count and all-cause and cardiovascular mortality: a meta-analysis. Eur J Prev Cardiol. 2023;30(18):1975–85 . 10.1093/eurjpc/zwad229 37555441

[3] Paluch AE, Bajpai S, Ballin M, et al. Prospective association of daily steps with cardiovascular disease: a harmonized meta-analysis. Circulation. 2023;147(2):122–31. 36537288 10.1161/CIRCULATIONAHA.122.061288 PMC9839547

[4] Paluch AE, Bajpai S, Bassett DR, et al. Daily steps and all-cause mortality: a meta-analysis of 15 international cohorts. Lancet Public Heal. 2022;7(3):e219–28. 10.1016/S2468-2667(21)00302-9 PMC9289978 35247352

[5] Master H, Annis J, Huang S, et al. Association of step counts over time with the risk of chronic disease in the all of Us research program. Nat Med. 2022;28(11):2301–8. 36216933 10.1038/s41591-022-02012-w PMC9671804

[6] Callisaya ML, Blizzard L, Schmidt MD, et al. Gait, gait variability and the risk of multiple incident falls in older people: a population-based study. Age Ageing. 2011;40(4):481–7. 21628390 10.1093/ageing/afr055

[7] Perera S, Patel KV, Rosano C, et al. Gait speed predicts incident disability: a pooled analysis. J Gerontol A Biol Sci Med Sci. 2015;71(1):63–71. 26297942 10.1093/gerona/glv126 PMC4715231

[8] Studenski S, Perera S, Patel K, et al. Gait speed and survival in older adults. J Am Med Assoc. 2011;305(1):50–8. 10.1001/jama.2010.1923 PMC3080184 21205966

[9] Tudor-Locke C, Bassett DR, Rutherford WJ, et al. BMI-referenced cut points for pedometer-determined steps per day in adults. J Phys Act Heal. 2008;5(SUPPL 1):126–39. 10.1123/jpah.5.s1.s126 PMC2866423 18364517

[10] Fan Y, Li Z, Han S, Lv C, Zhang B. The influence of gait speed on the stability of walking among the elderly. Gait Posture. 2016;47:31–6. 27264399 10.1016/j.gaitpost.2016.02.018

[11] Polhemus A, Ortiz LD, Brittain G, et al. Walking on common ground: a cross-disciplinary scoping review on the clinical utility of digital mobility outcomes. Npj Digit Med. 2021;4(1). 10.1038/s41746-021-00513-5. 10.1038/s41746-021-00513-5 PMC8516969 34650191

[12] Del Din S, Godfrey A, Galna B, Lord S, Rochester L. Free-living gait characteristics in ageing and Parkinson’s disease: impact of environment and ambulatory bout length. J Neuroeng Rehabil. 2016;13(1):1–12. 27175731 10.1186/s12984-016-0154-5 PMC4866360

[13] Buekers J, Megaritis D, Koch S, et al. Laboratory and free-living gait performance in adults with COPD and healthy controls. ERJ Open Res. 2023;00159:2023. 10.1183/23120541.00159-2023 PMC10518872 37753279

[14] Storm FA, Nair KPS, Clarke AJ, Van der Meulen JM, Mazzà C. Free-living and laboratory gait characteristics in patients with multiple sclerosis. PLoS ONE. 2018;13(5):1–15. 10.1371/journal.pone.0196463 PMC5929566 29715279

[15] Galperin I, Hillel I, Del Din S, et al. Associations between daily-living physical activity and laboratory-based assessments of motor severity in patients with falls and parkinson’s disease. Park Relat Disord. 2019;62(January):85–90. 10.1016/j.parkreldis.2019.01.022 30718220

[16] Shema-Shiratzky S, Hillel I, Mirelman A, et al. A wearable sensor identifies alterations in community ambulation in multiple sclerosis: contributors to real-world gait quality and physical activity. J Neurol. 2020;267(7):1912–21. 32166481 10.1007/s00415-020-09759-7

[17] Mc Ardle R, Taylor L, Cavadino A, Rochester L, Del Din S, Kerse N. Characterising walking behaviours in aged residential care using accelerometery: a cross-sectional comparison of care level, cognitive status and physical function (Preprint). JMIR Aging. 2023. 10.2196/53020. [Internet]. 10.2196/53020 PMC11185191 38842168

[18] Mc Ardle R, Sverdrup K, Del Din S, et al. Quantifying physical activity in aged residential care facilities: a structured review. Ageing Res Rev. 2021;67:101298. 33592308 10.1016/j.arr.2021.101298

[19] Donaire-Gonzalez D, Gimeno-Santos E, Balcells E, et al. Physical activity in COPD patients: patterns and bouts. Eur Respir J. 2013;42(4):993–1002. 23258786 10.1183/09031936.00101512

[20] Klenk J, Dallmeier D, Denkinger MD, et al. Objectively measured walking duration and sedentary behaviour and four-year mortality in older people. PLoS ONE. 2016;11(4):1–13. 10.1371/journal.pone.0153779 PMC4833405 27082963

[21] Rochester L, Mazzà C, Mueller A, et al. A roadmap to inform development, validation and approval of digital mobility outcomes: the mobilise-D approach. Digital Biomarkers. 2020;4(suppl 1):13–27. 33442578 10.1159/000512513 PMC7768123

[22] Micó-Amigo ME, Bonci T, Paraschiv-Ionescu A, et al. Assessing real-world gait with digital technology? Validation, insights and recommendations from the Mobilise-D consortium. J Neuroeng Rehabil. 2023;20(1):1–26. 37316858 10.1186/s12984-023-01198-5 PMC10265910

[23] Kirk C, Küderle A, Amigo MEM, et al. Mobilise – D insights to estimate real – world walking speed in multiple conditions with a wearable device. Sci Rep. 2024;14(1):1754. 10.1038/s41598-024-51766-5 PMC10799009 38243008

[24] Koch S, Buekers J, Cobo I, et al. From high-resolution time series to a single, clinically-interpretable value - considerations for the aggregation of real world walking speed assessed by wearable sensors in patients with chronic obstructive pulmonary disease (COPD). Eur Respir J. 2023;62(Suppl 67):PA1595.

[25] Mazzà C, Alcock L, Aminian K, et al. Technical validation of real-world monitoring of gait: a multicentric observational study. BMJ Open. 2021. 10.1136/bmjopen-2021-050785. 34857567 10.1136/bmjopen-2021-050785 PMC8640671

[26] Mikolaizak AS, Rochester L, Maetzler W, et al. Connecting real-world digital mobility assessment to clinical outcomes for regulatory and clinical endorsement–the Mobilise-D study protocol. PLoS ONE. 2022;17(10 October):1–21. 10.1371/journal.pone.0269615 PMC9536536 36201476

[27] Haley SM, Jette AM, Coster WJ, et al. Late life function and disability instrument: II. Development and evaluation of the function component. Journals Gerontol - Ser Biol Sci Med Sci. 2002;57(4):209–16. 10.1093/gerona/57.4.m217 11909886

[28] Miller MR, Hankinson J, Brusasco V, et al. Standardisation of spirometry. Eur Respir J. 2005;26(2):319–38. 16055882 10.1183/09031936.05.00034805

[29] JF K. Rating neurologic impairment in multiple sclerosis: an expanded disability status scale (EDSS). Neurology. Nov; 1983;33(11):1444–52. 6685237 10.1212/wnl.33.11.1444

[30] Goetz CG, Tilley BC, Shaftman SR, et al. Movement disorder society-sponsored revision of the unified parkinson’s disease rating scale (MDS-UPDRS): scale presentation and clinimetric testing results. Mov Disord. 2008;23(15):2129–70. 19025984 10.1002/mds.22340

[31] Guralnik JM, Simonsick EM, Ferrucci L, et al. A short physical performance battery assessing lower extremity function: association with Self-Reported disability and prediction of mortality and nursing home admission energetic cost of walking in older adults view project IOM committee on cognitive Agi. Artic J Gerontol. 1994;49(2):85–94. 10.1093/geronj/49.2.m85 8126356

[32] Demeyer H, Mohan D, Burtin C, et al. Chronic obstructive pulmonary diseases: objectively measured physical activity in patients with COPD : recommendations from an international task force on physical activity. J COPD Found. 2021;8(4):528–50. 10.15326/jcopdf.2021.0213 PMC8686852 34433239

[33] Niessen M, Pijnappels M, van Dieën J, van Lummel R. Detecting not-wearing periods during activity monitoring. Eur Respir Soc Annu Congr. 2013;2706(P4905):4905.

[34] McGraw KO, Wong SP. Forming inferences about some intraclass correlation coefficients. Psychol Methods. 1996;1(1):30–46.

[35] Koo TK, Li MY. A guideline of selecting and reporting intraclass correlation coefficients for reliability research. J Chiropr Med. 2016;15(2):155–63. 27330520 10.1016/j.jcm.2016.02.012 PMC4913118

[36] Baranowski T, Masse LC, Ragan B, Welk G. How many days was that? we’re still not Sure, but we’re asking the question Better! Med Sci Sport Exerc. 2008;40(7):S544–9. 10.1249/MSS.0b013e31817c6651 PMC2739114 18562972

[37] Scheers T, Philippaerts R, Lefevre J. Variability in physical activity patterns as measured by the sensewear armband: how many days are needed? Eur J Appl Physiol. 2012;112(5):1653–62. 21874552 10.1007/s00421-011-2131-9

[38] Matthews CE, Ainsworth BE, Thompson RW, Bassett DR. Sources of variance in daily physical activity levels as measured by an accelerometer. Med Sci Sports Exerc. 2002;34(8):1376–81. 12165695 10.1097/00005768-200208000-00021

[39] Hart TL, Swartz AM, Strath SJ. How many days of monitoring are needed to accurately estimate physical activity in older adults. Int J Behav Nutr Phys Act. 2011;8:62–9. 21679426 10.1186/1479-5868-8-62 PMC3130631

[40] Aadland E, Ylvisåker E. Reliability of objectively measured sedentary time and physical activity in adults. PLoS ONE. 2015;10(7):1–13. 10.1371/journal.pone.0133296 PMC4508000 26192184

[41] Matthews CE, Hagströmer M, Pober DM, Bowles HR. Best practices for using physical activity monitors in population-based research. Med Sci Sports Exerc. 2012;44(SUPPL 1):68–76. 10.1249/MSS.0b013e3182399e5b PMC3543867 22157777

[42] Penpraze V, Reilly JJ, MacLean CM, et al. Monitoring of physical activity in young children: how much is enough? Pediatr Exerc Sci. 2006;18(4):483–91. 39152609 10.1123/pes.18.4.483

[43] Mattocks C, Ness A, Leary S, et al. Use of accelerometers in a large field-based study of children: Protocols, design issues, and effects on precision. J Phys Act Heal. 2008;5(SUPPL. 1) 10.1123/jpah.5.s1.s98. 10.1123/jpah.5.s1.s98 18364528

[44] Hinkley T, O’Connell E, Okely AD, Crawford D, Hesketh K, Salmon J. Assessing volume of accelerometry data for reliability in preschool children. Med Sci Sports Exerc. 2012;44(12):2436–41. 22776873 10.1249/MSS.0b013e3182661478

[45] Trost SG, Pate RR, Freedson PS, Sallis JF, Taylor WC. Using objective physical activity measures with youth: how many days of monitoring are needed? Med Sci Sports Exerc. 2000;32(2):426–31. 10694127 10.1097/00005768-200002000-00025

[46] Antczak D, Lonsdale C, del Pozo Cruz B, Parker P, Sanders T. Reliability of geneactiv accelerometers to estimate sleep, physical activity, and sedentary time in children. Int J Behav Nutr Phys Act. 2021;18(1):1–11. 34090467 10.1186/s12966-021-01143-6 PMC8180134

[47] Motl RW, Zhu W, Park Y, McAuley E, Scott JA, Snook EM. Reliability of scores from physical activity monitors in adults with multiple sclerosis. Adapt Phys Activ Q. 2007;24(3):245–53. 17916920 10.1123/apaq.24.3.245

[48] Herrmann SD, Barreira TV, Kang M, Ainsworth BE. How many hours are enough? Accelerometer wear time may provide bias in daily activity estimates. J Phys Act Health. 2013;10(5):742–9. 23036822 10.1123/jpah.10.5.742

[49] Rich C, Geraci M, Griffiths L, Sera F, Dezateux C, Cortina-Borja M. Quality control methods in accelerometer data processing: defining minimum wear time. PLoS ONE. 2013;8(6):1–8. 10.1371/journal.pone.0067206 PMC3691227 23826236

