# Supplementary material for: Digital assessment of real-world walking in people with impaired mobility: How many hours and days are needed?
Source: Int J Behav Nutr Phys Act. 2025 Nov 21;22:148. doi: 10.1186/s12966-025-01851-3 (PMC12639672; doi:10.1186/s12966-025-01851-3)
Supplement: Supplementary file 1 — Supplementary Material 1. [file 12966_2025_1851_MOESM1_ESM.docx]

# Appendix 1: Recruitment sites and ethical approval

Appendix Table 1: Recruitment sites

| **Recruitment site** | **Condition(s)** | **Country** |
| --- | --- | --- |
| CAU Kiel (CAU) | PD and MS | Germany |
| CHU Montpellier (CHUM) | PFF | France |
| ICSTM London (ICL) | COPD | UK |
| IDSG Barcelona (ISG) | COPD | Spain |
| KUL Leuven (KUL1) | COPD | Belgium |
| KUL Leuven (KUL2) | PD | Belgium |
| Tel Aviv Sourasky Medical Center, Tel Aviv (TASMC) | PD | Israel |
| NTNU Trondheim (NTNU) | PFF | Norway |
| PFLC Grosshansdorf (PFLG) | COPD | Germany |
| RBGMF Stuttgart (RBMF) | PFF | Germany |
| TF Athens (TFG) | COPD | Greece |
| U Erlangen (UKER) | PD and MS | Germany |
| U Northumbria (UNN) | COPD | UK |
| U Sheffield (USFD) | MS | UK |
| Universität Zürich (UZH) | COPD | Switzerland |
| UNuT Newcastle (UNEW) | PD | UK |
| UVS San Raffaele (USR) | MS | Italy |

Ethical Commission (EC) of the Medical Faculty of Friedrich-Alexander University Erlangen-Nürnberg (Erlangen-Nürnberg, vote 535_20 B);

EC of the Medical Association of Schleswig-Holstein (Grosshansorf; vote 023/21 I);

EC of the Medical Faculty of Christian-Albrechts-University Kiel (Kiel; vote D 630/20);

EC Research of University Hospitals Leuven (vote S64977);

EC dell’Insubria (Milan; vote 196 del 2021);

Committee of the Protection of Persons, South-Mediterranean II (Montpellier; vote 221 B08);

EC of the Medical Faculty of Eberhard-Karls-University Tu¨bingen (Stuttgart; vote 976/2020BO2);

Helsinki Committee of the Tel Aviv Sourasky Medical Center (Tel Aviv, vote 0551-19-TLV 2020);

Regional Committee for Medical and Health Professional Research Ethics (Trondheim; vote 216069);

London-Bloomsbury Research Ethics Committee (all UK sites; vote 20/PR/0792);

Cantonal EC Zürich (Zurich; vote 2021–00601);

EC of Medical Research at Barcelona Hospital Clinic (Barcelona site 1; vote HCB/2021/0445), EC of Medical Research at Parc del Salud (Barcelona site 2; vote 2021/9797/I);

EC of Research at University Hospital Germans Trias i Pujol (Barcelona site 3; vote PI-21-093);

EC of Research at Viladecans Hospital (Barcelona site 4; vote PR129/21);

Sotiria Hospital Scientific Board (Athens; vote 1560/18-1-21).

# Appendix 2: Statistical analyses, model specifications and intraclass correlation coefficient (ICC) calculations

Participant characteristics, measurement characteristics and DMO values are presented as mean (SD) for normally distributed continuous variables, median (P25-P75 or P5-P95) for non-normally distributed continuous variables and as n (%) for categorical variables, using a complete-case approach.

Objectives 1-3 were addressed sequentially, and each DMO was separately analysed for each health condition (resulting in 24x4=96 DMO-condition combinations), as outlined below. The analyses for objective 1 assessed potential systematic measurement error of daily DMO values due to insufficient wear time, while objective 2-3 assessed potential random measurement error of weekly DMO values due to an insufficient number of valid measurement days (objective 2) or weekend days (objective 3).

As future studies will simultaneously examine multiple DMOs in multiple conditions (21), a separate processing pipeline for each of the 96 DMO-condition combinations in these studies is deemed impractical. Therefore, the results for all 96 DMO-condition combinations for objectives 1-3 were assessed by an expert group in order to propose DMO- and condition-agnostic recommendations for studies involving multiple walking activity and gait parameters or health conditions(objective 4). These recommendations seek to balance methodological rigour with analytical feasibility, and were based on the results of the 96 DMO-condition combinations, sample size, potential selection bias, random and systematic measurement errors, and clinical relevance. The expert group consisted of a multidisciplinary collaboration between clinical experts of the four conditions, experts on physical activity and gait, epidemiologists, statisticians, and engineers, making sure to account for all relevant technical, clinical and statistical considerations.

**Objective 1**

For objective 1, a linear (with or without log transformation, see below) mixed effects regression model was built for each DMO as the outcome (at the daily level), with a random intercept for participants, and daily wear time during waking hours (categorical) as exposure. As there are certain factors that could result in systematically higher or lower DMO values and wear time, the models were adjusted for relevant confounders (recruitment site, age, gender, height, BMI, years of education, employment status, living arrangement, number of prescribed medications, use of mobility aids indoors and outdoors, functional status, condition severity, wearable device type, number of measurement days of the participant, day number within assessment period (1-7), and season).

The minimum required daily wear time that constitutes a valid measurement day was determined for each DMO-condition combination as the upper limit of the highest wear time category with statistically significant differences in DMO values compared to the reference category. Then, DMO- and condition-agnostic recommendations on minimum wear time were proposed by expert consensus, taking into consideration sample size, potential selection bias, random measurement error, and systematic measurement error, and the clinical relevance of the different DMOs.

**Objective 2**

For objective 2, only measurement days that were considered valid, as determined by expert consensus in objective 1, were included. An unadjusted linear (with or without log transformation, Appendix 2) mixed effects regression model was built for each DMO as the outcome (at the daily level), with a random intercept for participants. As this objective addressed random measurement error, and not systematic measurement error as was done in objective 1, it was not required to adjust the models for any covariates.

Single-day within person variance ($\sigma_{e}^{2}$) and between person variance ($\sigma_{u}^{2}$) were directly extracted from the mixed effects regression models. Intraclass correlation coefficients (ICC) values for different numbers of measurement days (k) were then calculated, based on the ICC(k) formula from McGraw and Wong(34, 35), as follows:

For digital mobility outcomes (DMOs) that were not log transformed:

$$ICC\left( k \right)= \frac{\sigma_{u}^{2}}{\sigma_{u}^{2}+\frac{\sigma_{e}^{2}}{k}}$$

For DMOs that were log transformed:

$$ICC\left( k \right)= \frac{e^{\left( \sigma_{u}^{2} \right)}-1}{e^{\left( \sigma_{u}^{2}+\frac{\sigma_{e}^{2}}{k} \right)}-1}$$

The minimum required number of measurement days to obtain a reliable weekly DMO value for each DMO-condition combination was determined as the lowest number of days resulting in an ICC ≥0.80(36, 37), and a general recommendation was put forward by the experts group. This analysis was repeated after stratification by physical capacity (above or below condition-specific median SPPB score) as a post-hoc analysis.

**Example R code for the calculation of ICC values in objective 2 (using nlme package):**

# Mixed effects regression model

model <- lme(as.formula("DMO ~ 1"), random = ~ 1 | Participant_ID, data = data)

# [or for log transformed variables: model <- lme(as.formula("log(DMO) ~ 1"), random = ~ 1 | Participant_ID, data = data)]

# Extract between-person variances

between_var <- temp1[1,1] # stores between-participant variance

# Extract within-person variances

within_var <- getVarCov(model)[[1]] # stores within-participant variance

temp <- getVarCov(model, type = "conditional") # returns list

temp1 <- matrix(unlist(temp)) # converts list to matrix

# Calculate ICCs for k measurements days

icc <- c()

for (k in 1:7){

icc_1 <- within_var/(within_var+ between_var/k)

# [or for log transformed variables: icc_1 <- (exp(within_var)-1)/(exp(within_var + between_var/k)-1)]

icc <- c(icc, icc_1)

}

**Objective 3**

For objective 3, only valid measurement days were included. An unadjusted linear (with or without log transformation, Appendix 2) mixed effects regression model was built for each DMO as the outcome (at the daily level), with a random intercept for participants, and day type (week or weekend day) as exposure.

Single-day within person variance for weekdays ($\sigma_{e,week}^{2}$), single-day within person variance for weekend days ($\sigma_{e,weekend}^{2}$) and between person variance ($\sigma_{u}^{2}$) were directly extracted from the mixed effects regression models. Hereafter, ICC values for different numbers of weekdays (k_week_) and weekend days (k_weekend_) were then calculated as follows, but only for combinations for which the sum of week and weekend days equals the minimum required number of days determined by expert consensus in objective 2(34, 35):

For DMOs that were not log transformed:

$$ICC\left( k_{week}, k_{weekend} \right)= \frac{\sigma_{u}^{2}}{\sigma_{u}^{2}+\frac{{{(k}_{\mathrm{week}}* \sigma}_{e,week}^{2})+ {{(k}_{\mathrm{weekend}}* \sigma}_{e,weekend}^{2})}{{(k_{week}+k_{weekend})}^{2}}}$$

For DMOs that were log transformed:

$$ICC\left( k_{week}, k_{weekend} \right)= \frac{e^{\left( \sigma_{u}^{2} \right)}-1}{e^{\left( \sigma_{u}^{2}+\frac{{{(k}_{\mathrm{week}}* \sigma}_{e,week}^{2})+ {{(k}_{\mathrm{weekend}}* \sigma}_{e,weekend}^{2})}{{(k_{week}+k_{weekend})}^{2}} \right)}-1}$$

For each DMO-condition combination, the inclusion of weekend days was only required if the inclusion of weekend days resulted in an ICC ≥0.80 and including no weekend days in ICC <0.80. Then, the experts group proposed DMO- and condition-agnostic recommendations. This analysis was repeated after stratification by physical capacity (above or below condition-specific median SPPB score) as a post-hoc analysis.

**Example R code for the calculation of ICC values in objective 3 (using nlme package):**

# Mixed effects regression model

model <- lme(as.formula("DMO ~ day_type"), random = ~ 1 | Participant_ID, data = data, weights = varIdent(form = ~ 1 | day_type), method = "ML")

# [or for log transformed variables: model <- lme(as.formula("log(DMO) ~ day_type"), random = ~ 1 | Participant_ID, data = data, weights = varIdent(form = ~ 1 | day_type), method = "ML")]

# Different combinations of week and weekend days to test

prop <- c(3,3,3)

weekdays <- c(3,2,1)

weekends <- c(0,1,2)

# Extract between-person variances

varcov_matrix <- VarCorr(model) # variance matrix

between_var <- varcov_matrix[1, 1] # between-person variance

between_var <- as.numeric(between_var)

# Extract within-person variances

within_var <- summary(model)$sigma^2

weight0 <- 1 # weights for day_type=0 (weekday)

weight1 <- coef(model$modelStruct$varStruct, unconstrained=FALSE) # weigths for day_type=1 (weekend)

within_var0 <- (summary(model)$sigma*weight0)^2 # day_type=0 (weekday)

within_var1 <- (summary(model)$sigma*weight1)^2# day_type=1 (weekend)

# Calculate ICCs for 0, 1 or 2 weekend days:

icc <- c()

for (k in 1:3){

var_weekday <- c(var_weekday, within_var0*weekdays[k])

var_weekend <- c(var_weekend, within_var1*weekends[k])

within_var_b <- (var_weekday[k]+var_weekend[k])/(prop[k]^2)

icc_1 <- between_var/(between_var+within_var_b)

# [or for log transformed ariables: icc_1 <- (exp(between_var)-1)/(exp(between_var+within_var_b)-1)]

icc <- c(icc, icc_1)

}

Appendix Table 2: Overview of digital mobility outcomes (DMOs) that were log transformed for the analyses of Objectives 1, 2 and 3. The need for a log transformation was assessed based on a combination of the residual distribution (using Q-Q plots), heteroscedasticity of the residuals (plotting residuals against fitted values) and model fit (using Akaike information criteria and Bayesian information criteria) of the models using either the original or the log transformed variable.

| **Digital mobility outcome (DMO)** | **Log transformed?** |
| --- | --- |
| **Walking activity - Amount** | |
| Walking duration (h/day) | Yes |
| WB step count (steps/day) | Yes |
| **Walking activity - Pattern** | |
| Number of WB (WBs/day) | Yes |
| Number of WB >10s (WBs/day) | Yes |
| Number of WB >30s (WBs/day) | Yes |
| Number of WB >60s (WBs/day) | Yes |
| WB duration (s) | Yes |
| P90 WB duration (s) | Yes |
| WB duration variability (%) | Yes |
| **Gait - Pace** | |
| Walking speed in shorter (10-30s) WB (m/s) | No |
| Walking speed in longer (>30s) WB (m/s) | No |
| P90 walking speed in WB >10 s (m/s) | No |
| P90 walking speed in longer (>30s) WB (m/s) | No |
| Stride length in shorter (10-30s) WB (cm) | No |
| Stride length in longer (>30s) WB (cm) | No |
| **Gait - Rhythm** | |
| Cadence in all WB (steps/min) | No |
| Cadence in longer (>30s) WB (steps/min) | No |
| P90 cadence in longer (>30s) WB (steps/min) | No |
| Stride duration in all WB (s) | No |
| Stride duration in longer (>30s) WB (s) | No |
| **Gait – Bout to bout variability** | |
| Walking speed variability between longer (>30s) WB (%) | No |
| Stride length variability between longer (>30s) WB (%) | No |
| Cadence variability between all WB (%) | No |
| Stride duration variability between all WB (%) | No |

# Appendix 3: Tables

Appendix Table 3: Inclusion and exclusion criteria. COPD = chronic obstructive pulmonary disease; MS = multiple sclerosis; PD = Parkinson’s disease; PFF = proximal femoral fracture; WB = walking bout.

|  | **Inclusion** | **Exclusion** |
| --- | --- | --- |
| **All health conditions** | - Able to walk 4 meters independently with or without walking aids  - Anticipated availability for repeated study visits over 24 months  - Ability to consent and comply with any study specific procedures.  - Willingness to wear a wearable sensor for mobility monitoring  - Able to read and write in first language in the respective country | - Occurrence of any of the following within 3 months prior to informed consent: myocardial infarction, hospitalisation for unstable angina, stroke, coronary artery bypass graft, percutaneous coronary intervention, implantation of a cardiac resynchronisation therapy device, active treatment for cancer or other malignant disease, uncontrolled congestive heart disease (NYHA class >3), acute psychosis or major psychiatric disorders or continued substance abuse |
| **COPD** | - Aged 18 or over  - Diagnosis of COPD (post-bronchodilator forced expiratory volume in the first second (FEV_1_) to forced vital capacity ratio <0.70)  - Clinical stability, defined as at least 4 weeks after the onset of the last exacerbation  - Current or ex-smokers with a smoking history equivalent to at least 10 pack years (1 pack year = 20 cigarettes smoked per day for 1 year) | - Having undergone major lung surgery (e.g., lung transplant)  - Current diagnosis of lung cancer  - Primary respiratory diseases other than COPD  - Substantial limitations in mobility due to factors other than COPD  - Lung volume reduction within 6 months before inclusion |
| **MS** | - Aged 18 or over  - A diagnosis of MS based on the revised  McDonald’s criteria  - Expanded Disability Status Scale score of 3.0–6.5  - Clinical evidence of disability worsening over the previous two years | - Clinical relapse within 30 days prior to screening and baseline |
| **PD** | - Aged 18 or over  - Patients with the clinical diagnosis of PD  according to the recent criteria of the Movement Disorder Society  - Hoehn & Yahr stage I-III | - History consistent with Dementia with Lewy Bodies, atypical parkinsonian syndromes (including multiple system atrophy or progressive supranuclear palsy, diagnosed according to accepted criteria)  - Repeated strokes or stepwise progression of symptoms, leading to a diagnosis of ‘vascular  parkinsonism’  - Drug-induced Parkinsonism |
| **PFF** | - Aged 45 or over  - Surgical treatment (fixation or arthroplasty) for a low-energy fracture of the proximal femur (ICD-10 diagnosis S72.0, S72.1, S72.2) as diagnosed on X-rays of the hip and pelvis  - Between 3 days and 52 weeks post-surgery | - Not able to walk before treatment of hip fracture |

|  | **Source DMO at WB level** | **Daily level** | **Weekly level** |
| --- | --- | --- | --- |
| **Walking activity – Amount** | | | |
| Walking duration (h/day) | WB duration | Daily sum of WB duration using all WBs | Weekly mean of daily sum of WB duration using all WBs |
| WB step count (steps/day) | Steps | Sum of steps using all WBs | Weekly mean of daily sum of steps using all WBs |
| **Walking activity - Pattern** | | | |
| Number of WBs (WBs/day) | Number of WBs | Daily sum of all WBs | Weekly mean of daily sum of all WBs |
| Number of WBs >10s (WBs/day) | Number of WBs | Daily sum of number of WBs longer than 10s | Weekly mean of daily sum of number of WBs longer than 10s |
| Number of WBs >30s (WBs/day) | Number of WBs | Daily sum of number of WBs longer than 30s | Weekly mean of daily sum of number of WBs longer than 30s |
| Number of WBs >60s (WBs/day) | Number of WBs | Daily sum of number of WBs longer than 60s | Weekly mean of daily sum of number of WBs longer than 60s |
| WB duration (s) | WB duration | Daily median of WB duration using all WBs | Weekly mean of daily median of WB duration using all WBs |
| P90 WB duration (s) | WB duration | Daily P90 of WB duration using all WBs | Weekly mean of daily P90 of WB duration using all WBs |
| WB duration bout to bout variability (%) | WB duration | Daily COV of WB duration using all WBs | Weekly mean of daily COV of WB duration using all WBs |
| **Gait - Pace** | | | |
| Walking speed in shorter (10-30s) WBs (m/s) | Walking speed | Daily mean of walking speed using WBs between 10s and 30s | Weekly mean of daily mean walking speed using WBs between 10s and 30s |
| Walking speed in longer (>30s) WBs (m/s) | Walking speed | Daily mean of walking speed using WBs longer than 30s | Weekly mean of daily mean walking speed using WBs longer than 30s |
| P90 walking speed in WBs >10 s (m/s) | Walking speed | Daily P90 of walking speed using WBs longer than 10s | Weekly mean of daily P90 of walking speed using WBs longer than 10s |
| P90 walking speed in longer (>30s) WBs (m/s) | Walking speed | Daily P90 of walking speed using WBs longer than 30s | Weekly mean of daily P90 of walking speed using WBs longer than 30s |
| Stride length in shorter (10-30s) WBs (cm) | Stride length | Daily mean of stride length using WBs between 10s and 30s | Weekly mean of daily mean of stride length using WBs between 10s and 30s |
| Stride length in longer (>30s) WBs (cm) | Stride length | Daily mean of stride length using WBs longer than 30s | Weekly mean of daily mean of stride length using WBs longer than 30s |
| **Gait - Rhythm** | | | |
| Cadence in all WBs (steps/min) | Cadence | Daily mean of cadence using all WBs | Weekly mean of daily mean of cadence using all WBs |
| Cadence in longer (>30s) WBs (steps/min) | Cadence | Daily mean of cadence using WBs longer than 30s | Weekly mean of daily mean of cadence using WBs longer than 30s |
| P90 cadence in longer (>30s) WBs (steps/min) | Cadence | Daily P90 using WBs longer than 30s | Weekly mean of daily P90 using WBs longer than 30s |
| Stride duration in all WBs (s) | Stride duration | Daily mean of stride duration using all WBs | Weekly mean of daily mean of stride duration using all WBs |
| Stride duration in longer (>30s) WBs (s) | Stride duration | Daily mean of stride duration using WBs longer than 30s | Weekly mean of daily mean of stride duration using WBs longer than 30s |
| **Gait – Bout to bout variability** | | | |
| Walking speed bout to bout variability between longer (>30s) WBs (%) | Walking speed | Daily COV of walking speed using WBs longer than 30s | Weekly mean of daily COV of walking speed using WBs longer than 30s |
| Stride length bout to bout variability between longer (>30s) WBs (%) | Stride length | Daily COV of stride length using WBs longer than 30s | Weekly mean of daily COV of stride length using WBs longer than 30s |
| Cadence bout to bout variability (%) | Cadence | Daily COV using all WBs | Weekly mean of daily COV using all WBs |
| Stride duration bout to bout variability (%) | Stride duration | Daily COV of stride duration using all WBs | Weekly mean of daily COV of stride duration using all WBs |

Appendix Table 4: Definition of digital mobility outcomes (DMOs) at the walking bout (WB), daily and weekly level, organised in five walking domains. COPD = chronic obstructive pulmonary disease; MS = multiple sclerosis; PD = Parkinson’s disease; PFF = proximal femoral fracture; COV = coefficient of variance.

Appendix Table 5: Characteristics of Mobilise-D clinical validation study participants that were excluded or included in the present analysis (“n” refers to the number of participants). P-values are based on Student's t-test or Wilcoxon rank-sum test for continuous variables and Fisher's exact test for proportions. Values in bold indicate statistically significant differences (p < 0.05).

|  | **COPD**  **(n=606)** | | | **MS**  **(n=602)** | | | **PD**  **(n=600)** | | | **PFF**  **(n=568)** | | |
| --- | --- | --- | --- | --- | --- | --- | --- | --- | --- | --- | --- | --- |
|  | Included  (n=565) | Excluded  (n=41) | p-value | Included  (n=558) | Excluded  (n=44) | p-value | Included  (n=543) | Excluded  (n=57) | p-value | Included  (n=487) | Excluded  (n=81) | p-value |
| **Age** (years), mean (SD) | 68 (8) | 68 (8) | 0.76 | 52 (11) | 54 (12) | 0.26 | 66 (10) | 65 (9) | 0.68 | 77 (10) | 78 (10) | 0.46 |
| **Gender: Female**, n (%) | 206 (36) | 15 (37) | 1.00 | 358 (64) | 29 (66) | 0.87 | 197 (36) | 14 (25) | 0.08 | 319 (66) | 57 (70) | 0.45 |
| **Height** (cm), mean (SD) | 168 (9) | 168 (8) | 0.56 | 170 (9) | 170 (9) | 0.46 | 172 (10) | 174 (8) | 0.30 | 168 (10) | 168 (10) | 0.78 |
| **Body mass index** (kg/m^2^), mean (SD) | 27 (5) | 27 (5) | 0.82 | 26 (6) | 26 (6) | 0.48 | 26 (4) | 26 (4) | 0.76 | 24 (4) | 24 (5) | 0.50 |
| **Years of education**, median (P25-P75) | 13 (11-15) | 14 (11-16) | 0.44 | 15 (12-18) | 15(11-17) | 0.95 | 16 (13-18) | 16 (14-17) | 0.44 | 13 (10-15) | 12 (9-15) | 0.15 |
| **Number of prescribed medications**, median (P25-P75) | 7 (4-9) | 6 (3-10) | 0.42 | 4 (2-6) | 5 (3-7) | 0.17 | 5 (3-7) | 5 (3-7) | 0.87 | **7 (4-10)** | **9 (5-11)** | **0.033** |
| **Use of mobility aids indoor**, n (%) | 12 (2) | 2 (5) | 0.24 | **134 (24)** | **18 (41)** | **0.018** | 19 (3) | 3 (5) | 0.46 | 262 (54) | 38 (48) | 0.33 |
| **Use of mobility aids outdoor**, n (%) | 49 (9) | 5 (12) | 0.40 | 327 (59) | 29 (66) | 0.43 | 56 (10) | 5 (9) | 1.00 | 327 (67) | 49 (62) | 0.37 |
| **LLFDI-FC**, mean (SD) | 59 (9) | 60 (11) | 0.35 | **53 (12)** | **48 (13)** | **0.005** | 63 (12) | 62 (10) | 0.59 | 53 (13) | 54 (14) | 0.37 |
| **FEV_1_** (%predicted), mean (SD) | **54 (20)** | **60 (15)** | **0.03** |  |  |  |  |  |  |  |  |  |
| **EDSS** score, median (P25-P75) |  |  |  | **4.5 (1.2)** | **5.5 (1.2)** | **0.006** |  |  |  |  |  |  |
| **MDS-UPDRS III**, mean (SD) |  |  |  |  |  |  | 26 (12) | 27 (13) | 0.44 |  |  |  |
| **SPPB** (total score), mean (SD) | 9.9 (1.8) | 10.1 (1.6) | 0.78 | **7.7 (2.9)** | **5.8 (3.5)** | **0.001** | **9.7 (1.7)** | **9.0 (1.8)** | **0.003** | **6.4 (3.1)** | **4.6 (3.4)** | **< 0.001** |

COPD = chronic obstructive pulmonary disease; MS = multiple sclerosis; PD = Parkinson’s disease; PFF = proximal femoral fracture; LLFDI-FC = Functional Component of the Late Life Function and Disability Instrument; FEV1 = forced expiratory volume in the first second; EDSS = Expanded Disability Status Scale; MDS-UPDRS = Movement Disorder Society – Unified Parkinson’s Disease Rating Scale III; SPPB = Short Physical Performance Battery.

Appendix Table 6: Characteristics for participants included in the present analysis who reached (i.e., included) or did not reach (i.e., excluded) general recommendations of having >12h of measurements for ≥3 days. P-values are based on Student's t-test or Wilcoxon rank-sum test for continuous variables and Fisher's exact test for proportions. Values in bold indicate statistically significant differences (p < 0.05).

|  | **COPD**  **(n=565)** | | | **MS**  **(n=558)** | | | **PD**  **(n=543)** | | | **PFF**  **(n=487)** | | |
| --- | --- | --- | --- | --- | --- | --- | --- | --- | --- | --- | --- | --- |
|  | Included  (n=538) | Excluded  (n=27) | p-value | Included  (n=517) | Excluded  (n=41) | p-value | Included  (n=522) | Excluded  (n=21) | p-value | Included  (n=412) | Excluded  (n=75) | p-value |
| **Age** (years), mean (SD) | 67 (8) | 70 (10) | 0.12 | 52 (11) | 49 (13) | 0.86 | 66 (10) | 65 (10) | 0.71 | **77 (10)** | **80 (8)** | **0.003** |
| **Gender: Female**, n (%) | 198 (37) | 8 (30) | 0.54 | 332 (64) | 26 (63) | 1.00 | 188 (36) | 9 (43) | 0.64 | **254 (63)** | **60 (80)** | **0.004** |
| **Height** (cm), mean (SD) | 168 (9) | 170 (8) | 0.70 | 170 (9) | 171 (9) | 0.48 | 172 (10) | 171 (9) | 0.51 | **168 (10)** | **164 (9)** | **< 0.001** |
| **Body mass index** (kg/m^2^), mean (SD) | 27 (5) | 26 (9) | 0.48 | **27 (6)** | **25 (6)** | **0.01** | 27 (4) | 25 (4) | 0.053 | 24 (4) | 24 (4) | 0.83 |
| **Years of education**, median (P25-P75) | **13 (11-15)** | **12 (8-13)** | **0.04** | 15 (12-18) | 15 (12-17) | 0.85 | 16 (13-18) | 16 (14-17) | 0.94 | 13 (10-15) | 12 (9-15) | 0.05 |
| **Number of prescribed medications**, median (P25-P75) | 6 (4-9) | 6 (5-7) | 0.49 | 4 (2-6) | 4 (2-5) | 0.74 | 5 (3-7) | 5 (3-5) | 0.63 | **7 (3-10)** | **9 (6-11)** | **0.01** |
| **Use of mobility aids indoor**, n (%) | 10 (2) | 2 (7) | 0.11 | **110 (21)** | **24 (59)** | **< 0.001** | 19 (4) | 0 (0) | 0.17 | 222 (54) | 47 (52) | 1.00 |
| **Use of mobility aids outdoor**, n (%) | **41 (8)** | **8 (30)** | **0.001** | **293 (57)** | **34 (83)** | **< 0.001** | 54 (10) | 2 (10) | 1.00 | 278 (67) | 59 (65) | 0.79 |
| **LLFDI-FC**, mean (SD) | **59 (9)** | **54 (8)** | **0.004** | **53 (12)** | **47 (9)** | **0.003** | **64 (12)** | **57 (8)** | **0.009** | **54 (13)** | **51 (13)** | **0.02** |
| **FEV_1_** (%predicted), mean (SD) | 54 (20) | 51 (18) | 0.74 |  |  |  |  |  |  |  |  |  |
| **EDSS** score, median (P25-P75) |  |  |  | **5 (4-6)** | **6.5 (6-6.5)** | **< 0.001** |  |  |  |  |  |  |
| **MDS-UPDRS III**, mean (SD) |  |  |  |  |  |  | 26 (12) | 31 (16) | 0.19 |  |  |  |
| **SPPB** (total score), mean (SD) | **10.1 (1.6)** | **8.9 (2.2)** | **0.003** | **7.9 (2.7)** | **4.9 (3.5)** | **< 0.001** | 9.7 (1.7) | 9.3 (2.1) | 0.67 | **6.8 (3.1)** | **4.1 (2.3)** | **< 0.001** |
| **Device type:**  McRoberts MoveMonitor+, n (%)  Axivity AX6, n (%) | 443 (82)  95 (18) | 25 (93)  2 (7) | 0.29 | **86 (17)**  **431 (83)** | **31 (76)**  **10 (24)** | **0.02** | 367 (70)  155 (30) | 19 (90)  2 (10) | 0.05 | **83 (20)**  **329 (80)** | **29 (39)**  **46 (61)** | **< 0.001** |
| **Measurement days** (days), median (P25-P75) | **7 (7-7)** | **5 (3-7)** | **< 0.001** | **7 (7-7)** | **6 (6-7)** | **< 0.001** | **7 (7-7)** | **4 (2-6)** | **< 0.001** | **7 (7-7)** | **6 (6-7)** | **< 0.001** |

COPD = chronic obstructive pulmonary disease; MS = multiple sclerosis; PD = Parkinson’s disease; PFF = proximal femoral fracture; LLFDI-FC = Functional Component of the Late Life Function and Disability Instrument; FEV1 = forced expiratory volume in the first second; EDSS = Expanded Disability Status Scale; MDS-UPDRS = Movement Disorder Society – Unified Parkinson’s Disease Rating Scale III; SPPB = Short Physical Performance Battery.

Appendix Table 7: Ranges of Digital Mobility Outcome (DMO) values at the day level of the 2153 included participants (“n” refers to the number of days).

|  | All  (n = 13196)) |
| --- | --- |
| **Walking activity - Amount** | |
| **Walking duration** (h/day), min-max | 0.01-8.02 |
| **WB step count** (steps/day), min-max | 39-38858 |
| **Walking activity – Pattern** | |
| **Number of WBs** (WBs/day), min-max | 2-1553 |
| **Number of WBs >10s** (WBs/day), min-max | 1-714 |
| **Number of WBs >30s** (WBs/day), min-max | 1-193 |
| **Number of WBs >60s** (WBs/day), min-max | 0-95 |
| **WB duration** (s), min-max | 4.6-38.4 |
| **P90 WB duration** (s), min-max | 9.2-298.2 |
| **WB duration bout to bout variability** (%), min-max | 40-142 |
| **Gait - Pace** | |
| **Walking speed in shorter (10-30s) WBs** (m/s), min-max | 0.3-1.3 |
| **Walking speed in longer (>30s) WBs** (m/s), min-max | 0.3-1.8 |
| **P90 walking speed in WBs >10 s** (m/s), min-max | 0.3-1.8 |
| **P90 walking speed in longer (>30s) WBs** (m/s), min-max | 0.3-2.0 |
| **Stride length in shorter (10-30s) WBs** (cm), min-max | 49-168 |
| **Stride length in longer (>30s)** **WBs** (cm), min-max | 47-234 |
| **Gait – Rhythm** | |
| **Cadence in all WBs** (steps/min), min-max | 61-111 |
| **Cadence in longer (>30s) WBs** (steps/min), min-max | 55-124 |
| **P90 cadence in longer (>30s) WBs** (steps/min), min-max | 55-130 |
| **Stride duration in all WBs** (s), min-max | 0.73-1.94 |
| **Stride duration in longer** (>30s) WB (s), min-max | 0.67-2.02 |
| **Gait – Bout to bout variability** | |
| **Walking speed bout to bout variability between longer (>30s) WBs** (-),min-max | 0-76 |
| **Stride length bout to bout variability between longer (>30s) WBs** (-),min-max | 0-70 |
| **Cadence bout to bout variability** (-),min-max | 4-29 |
| **Stride duration bout to bout variability** (-),min-max | 6-40 |

COPD = chronic obstructive pulmonary disease; MS = multiple sclerosis; PD = Parkinson’s disease; PFF = proximal femoral fracture; WB = walking bout.

Appendix Table 8: Number of participants that reached different combinations of minimum daily wear time and minimum number of days thresholds (“n” refers to number of participants) out of 2153 participants included in the present study.

|  | **≥1 days** | **≥2 days** | **≥3 days** | **≥4 days** | **≥5 days** | **≥6 days** | **≥7 days** |
| --- | --- | --- | --- | --- | --- | --- | --- |
| **>12h**, n (%) | 2127 (99) | 2059 (96) | 1989 (92) | 1899 (88) | 1778 (83) | 1585 (74) | 1205 (56) |
| **>13h**, n (%) | 2119 (98) | 2049 (95) | 1975 (92) | 1872 (87) | 1751 (81) | 1542 (72) | 1117 (52) |
| **≥14h**, n (%) | 2102 (98) | 2023 (94) | 1934 (90) | 1806 (84) | 1656 (77) | 1385 (64) | 889 (41) |

Appendix Table 9: Minimum required number of measurement days for the 192 digital mobility outcome (DMO)-condition-physical capacity combinations, after stratifying according to physical capacity (below (lower) or above (higher) condition-specific median Short Physical Performance Battery (SPPB) score). A forward slash (/) indicates that one week of measurements was insufficient to reach an intraclass correlation coefficient ≥ 0.80. * indicates domains that are yet to be clinically validated.

|  | | COPD | | MS | | PD | | PFF |
| --- | --- | --- | --- | --- | --- | --- | --- | --- |
| **Number of participants**  Lower SPPB  Higher SPPB | | 253  301 | | 297  261 | | 355  185 | | 262  225 |
| **Walking activity - Amount** | | | | | | | | |
| **Walking duration**  Lower SPPB  Higher SPPB | ≥2  ≥3 | | ≥2  ≥4 | | ≥3  ≥5 | | ≥3  ≥3 | |
| **WB step count**  Lower SPPB  Higher SPPB | ≥2  ≥3 | | ≥2  ≥3 | | ≥3  ≥5 | | ≥3  ≥2 | |
| **Walking activity – Pattern*** | | | | | | | | |
| **Number of WBs**  Lower SPPB  Higher SPPB | | ≥2  ≥2 | | ≥2  ≥3 | | ≥2  ≥3 | | ≥2  ≥2 |
| **Number of WBs >10s**  Lower SPPB  Higher SPPB | | ≥2  ≥2 | | ≥2  ≥3 | | ≥2  ≥3 | | ≥3  ≥2 |
| **Number of WBs >30s**  Lower SPPB  Higher SPPB | | ≥4  ≥4 | | ≥6  ≥6 | | ≥5  ≥7 | | ≥6  ≥7 |
| **Number of WBs >60s**  Lower SPPB  Higher SPPB | | ≥6  ≥6 | | /  / | | /  / | | ≥6  / |
| **WB duration**  Lower SPPB  Higher SPPB | | ≥3  ≥3 | | ≥3  ≥5 | | ≥4  ≥4 | | ≥4  ≥3 |
| **P90 WB duration**  Lower SPPB  Higher SPPB | | ≥5  ≥4 | | ≥3  / | | ≥6  / | | ≥4  ≥4 |
| **WB duration bout to bout variability**  Lower SPPB  Higher SPPB | | ≥5  ≥4 | | ≥7  ≥6 | | ≥7  / | | ≥4  ≥6 |
| **Gait - Pace** | | | | | | | | |
| **Walking speed in shorter (10-30s) WBs**  Lower SPPB  Higher SPPB | | ≥1  ≥1 | | ≥1  ≥2 | | ≥2  ≥2 | | ≥2  ≥1 |
| **Walking speed in longer (>30s) WBs**  Lower SPPB  Higher SPPB | | ≥3  ≥3 | | ≥2  ≥3 | | ≥3  ≥4 | | ≥3  ≥2 |
| **P90 walking speed in WBs >10 s**  Lower SPPB  Higher SPPB | | ≥2  ≥2 | | ≥1  ≥2 | | ≥2  ≥2 | | ≥2  ≥1 |
| **P90 walking speed in longer (>30s) WBs**  Lower SPPB  Higher SPPB | | ≥2  ≥3 | | ≥2  ≥2 | | ≥2  ≥3 | | ≥3  ≥2 |
| **Stride length in shorter (10-30s) WBs**  Lower SPPB  Higher SPPB | | ≥1  ≥1 | | ≥2  ≥2 | | ≥1  ≥2 | | ≥1  ≥1 |
| **Stride length in longer (>30s)** **WBs**  Lower SPPB  Higher SPPB | | ≥2  ≥3 | | ≥2  ≥4 | | ≥2  ≥3 | | ≥2  ≥2 |
| **Gait – Rhythm** | | | | | | | | |
| **Cadence in all WBs**  Lower SPPB  Higher SPPB | | ≥1  ≥1 | | ≥1  ≥1 | | ≥1  ≥1 | | ≥1  ≥1 |
| **Cadence in longer (>30s) WBs**  Lower SPPB  Higher SPPB | | ≥2  ≥2 | | ≥2  ≥3 | | ≥3  ≥3 | | ≥3  ≥2 |
| **P90 cadence in longer (>30s) WBs**  Lower SPPB  Higher SPPB | | ≥2  ≥3 | | ≥2  ≥2 | | ≥3  ≥3 | | ≥3  ≥2 |
| **Stride duration in all WBs**  Lower SPPB  Higher SPPB | | ≥2  ≥1 | | ≥2  ≥3 | | ≥2  ≥3 | | ≥2  ≥2 |
| **Stride duration in longer (>30s) WBs**  Lower SPPB  Higher SPPB | | ≥3  ≥3 | | ≥3  ≥4 | | ≥4  ≥4 | | ≥3  ≥2 |
| **Gait – Bout to bout variability*** | | | | | | | | |
| **Walking speed bout to bout variability between longer (>30s) WBs**  Lower SPPB  Higher SPPB | | ≥6  ≥7 | | ≥6  ≥7 | | ≥7  / | | /  ≥7 |
| **Stride length bout to bout variability between longer (>30s) WBs**  Lower SPPB  Higher SPPB | | ≥7  / | | ≥6  ≥7 | | /  / | | ≥7  / |
| **Cadence bout to bout variability**  Lower SPPB  Higher SPPB | | ≥2  ≥2 | | ≥2  ≥3 | | ≥2  ≥3 | | ≥4  ≥2 |
| **Stride duration bout to bout variability**  Lower SPPB  Higher SPPB | | ≥4  ≥4 | | ≥3  ≥6 | | ≥7  ≥6 | | ≥6  ≥3 |

COPD = chronic obstructive pulmonary disease; MS = multiple sclerosis; PD = Parkinson’s disease; PFF = proximal femoral fracture; WB = walking bout. SPPB score is missing for 11 people with COPD and 3 people with PD.

Appendix Table 10: Effect of different combinations in number of week and weekend days on intraclass correlation coefficient (ICC) values when 3 measurement days are considered (green cell indicates ICC ≥0.80; 0 = 0 weekend days and 3 weekday; 1 = 1 weekend day and 2 weekdays; 2 = 2 weekend days and 1 weekday).

|  | COPD  (n = 565) | | | MS  (n = 558) | | | PD  (n = 543) | | | | PFF  (n = 487) | | | | | |  |
| --- | --- | --- | --- | --- | --- | --- | --- | --- | --- | --- | --- | --- | --- | --- | --- | --- | --- |
| **Number of weekend days** | 0 | 1 | 2 | 0 | 1 | 2 | 0 | 1 | 2 | | 0 | | 1 | | 2 | |  |
| **Walking activity - Amount** | | | | | | | | | | | | | | | | | |
| **Walking duration** | 0.90 | 0.88 | 0.87 | 0.89 | 0.88 | 0.86 | 0.82 | 0.80 | 0.78 | | 0.88 | | 0.89 | | 0.90 | |  |
| **WB step count** | 0.90 | 0.89 | 0.87 | 0.89 | 0.88 | 0.87 | 0.82 | 0.80 | 0.77 | | 0.89 | | 0.90 | | 0.91 | |  |
| **Walking activity – Pattern** | | | | | | | | | | | | | | | | | |
| **Number of WBs** | 0.91 | 0.89 | 0.88 | 0.91 | 0.90 | 0.89 | 0.87 | 0.85 | 0.83 | | 0.93 | | 0.93 | | 0.93 | |  |
| **Number of WBs >10s** | 0.91 | 0.90 | 0.89 | 0.90 | 0.89 | 0.88 | 0.87 | 0.84 | 0.82 | | 0.88 | | 0.89 | | 0.89 | |  |
| **Number of WBs >30s** | 0.80 | 0.78 | 0.77 | 0.73 | 0.71 | 0.69 | 0.71 | 0.67 | 0.63 | | 0.69 | | 0.70 | | 0.72 | |  |
| **Number of WBs >60s** | 0.69 | 0.67 | 0.66 | 0.63 | 0.60 | 0.58 | 0.58 | 0.55 | 0.52 | | 0.59 | | 0.61 | | 0.63 | |  |
| **WB duration** | 0.84 | 0.83 | 0.83 | 0.79 | 0.78 | 0.77 | 0.80 | 0.79 | 0.77 | | 0.83 | | 0.82 | | 0.82 | |  |
| **P90 WB duration** | 0.76 | 0.75 | 0.75 | 0.76 | 0.74 | 0.71 | 0.68 | 0.66 | 0.64 | | 0.75 | | 0.77 | | 0.79 | |  |
| **WB duration bout to bout variability** | 0.76 | 0.75 | 0.74 | 0.72 | 0.71 | 0.70 | 0.65 | 0.62 | 0.59 | | 0.70 | | 0.71 | | 0.73 | |  |
| **Gait - Pace** | | | | | | | | | | | | | | | | | |
| **Walking speed in shorter (10-30s) WBs** | 0.95 | 0.950 | 0.94 | 0.93 | 0.93 | 0.92 | 0.92 | 0.92 | 0.91 | 0.96 | | 0.96 | | 0.96 | |  |  |
| **Walking speed in longer (>30s) WBs** | 0.86 | 0.85 | 0.85 | 0.90 | 0.90 | 0.89 | 0.85 | 0.83 | 0.81 | 0.93 | | 0.93 | | 0.93 | |  |  |
| **P90 walking speed in WBs >10 s** | 0.90 | 0.90 | 0.90 | 0.95 | 0.94 | 0.94 | 0.92 | 0.91 | 0.90 | 0.96 | | 0.96 | | 0.96 | |  |  |
| **P90 walking speed in longer (>30s) WBs** | 0.88 | 0.87 | 0.87 | 0.93 | 0.92 | 0.92 | 0.87 | 0.85 | 0.84 | 0.94 | | 0.94 | | 0.94 | |  |  |
| **Stride length in shorter (10-30s) WBs** | 0.96 | 0.96 | 0.96 | 0.92 | 0.91 | 0.90 | 0.94 | 0.93 | 0.93 | 0.95 | | 0.95 | | 0.95 | |  |  |
| **Stride length in longer (>30s)** **WBs** | 0.87 | 0.87 | 0.87 | 0.88 | 0.87 | 0.85 | 0.88 | 0.86 | 0.85 | 0.93 | | 0.92 | | 0.92 | |  |  |
| **Gait – Rhythm** | | | | | | | | | | | | | | | | | |
| **Cadence in all WBs** | 0.96 | 0.96 | 0.96 | 0.97 | 0.96 | 0.96 | 0.95 | 0.95 | 0.95 | 0.97 | | 0.97 | | 0.97 | |  |  |
| **Cadence in longer (>30s) WBs** | 0.88 | 0.88 | 0.88 | 0.91 | 0.91 | 0.91 | 0.85 | 0.83 | 0.80 | 0.92 | | 0.91 | | 0.91 | |  |  |
| **P90 cadence in longer (>30s) WBs** | 0.88 | 0.88 | 0.87 | 0.92 | 0.92 | 0.91 | 0.85 | 0.82 | 0.79 | 0.92 | | 0.91 | | 0.91 | |  |  |
| **Stride duration in all WBs** | 0.93 | 0.92 | 0.92 | 0.88 | 0.87 | 0.86 | 0.87 | 0.86 | 0.84 | 0.90 | | 0.90 | | 0.91 | |  |  |
| **Stride duration in longer (>30s) WBs** | 0.86 | 0.86 | 0.85 | 0.85 | 0.84 | 0.83 | 0.82 | 0.80 | 0.78 | 0.85 | | 0.85 | | 0.86 | |  |  |
| **Gait – Bout to bout variability** | | | | | | | | | | | | | | | | | |
| **Walking speed bout to bout variability between longer (>30s) WBs** | 0.71 | 0.69 | 0.68 | 0.72 | 0.70 | 0.68 | 0.66 | 0.63 | 0.60 | 0.66 | | 0.66 | | 0.67 | |  |  |
| **Stride length bout to bout variability between longer (>30s) WBs** | 0.66 | 0.63 | 0.61 | 0.69 | 0.67 | 0.65 | 0.59 | 0.58 | 0.56 | 0.65 | | 0.65 | | 0.65 | |  |  |
| **Cadence bout to bout variability** | 0.88 | 0.88 | 0.87 | 0.86 | 0.86 | 0.86 | 0.87 | 0.87 | 0.87 | 0.84 | | 0.84 | | 0.84 | |  |  |
| **Stride duration bout to bout variability** | 0.80 | 0.79 | 0.77 | 0.79 | 0.77 | 0.76 | 0.69 | 0.66 | 0.64 | 0.75 | | 0.76 | | 0.76 | |  |  |

COPD = chronic obstructive pulmonary disease; MS = multiple sclerosis; PD = Parkinson’s disease; PFF = proximal femoral fracture; WB = walking bout.

*Appendix Table 11: Effect of different combinations in number of week and weekend days on intraclass correlation coefficient (ICC) values when 3 measurement days are considered, after stratifying according to physical capacity (below (lower) or above (higher) condition-specific median Short Physical Performance Battery (SPPB) score). Green cell indicates ICC ≥0.80 (0 = 0 weekend days and 3 weekday; 1 = 1 weekend day and 2 weekdays; 2 = 2 weekend days and 1 weekday).*

|  | COPD | | | MS | | | PD | | | | PFF | | | | | |  |
| --- | --- | --- | --- | --- | --- | --- | --- | --- | --- | --- | --- | --- | --- | --- | --- | --- | --- |
| **Number of participants**  Lower SPPB  Higher SPPB | 253  301 | | | 297  261 | | | 355  185 | | | | 262  225 | | | | | |  |
| **Number of weekend days** | 0 | 1 | 2 | 0 | 1 | 2 | 0 | 1 | 2 | | 0 | | 1 | | 2 | |  |
| **Walking activity - Amount** | | | | | | | | | | | | | | | | | |
| **Walking duration** |  |  |  |  |  |  |  |  |  | |  | |  | |  | |  |
| Lower SPPB | 0.90 | 0.89 | 0.88 | 0.90 | 0.89 | 0.88 | 0.85 | 0.82 | 0.80 | | 0.83 | | 0.84 | | 0.85 | |  |
| Higher SPPB | 0.87 | 0.86 | 0.84 | 0.81 | 0.79 | 0.78 | 0.76 | 0.74 | 0.72 | | 0.83 | | 0.84 | | 0.86 | |  |
| **WB step count** |  |  |  |  |  |  |  |  |  | |  | |  | |  | |  |
| Lower SPPB | 0.90 | 0.89 | 0.89 | 0.91 | 0.90 | 0.89 | 0.84 | 0.82 | 0.80 | | 0.84 | | 0.85 | | 0.86 | |  |
| Higher SPPB | 0.87 | 0.86 | 0.84 | 0.82 | 0.80 | 0.78 | 0.75 | 0.72 | 0.70 | | 0.85 | | 0.86 | | 0.87 | |  |
| **Walking activity – Pattern** | | | | | | | | | | | | | | | | | |
| **Number of WBs** |  |  |  |  |  |  |  |  |  | |  | |  | |  | |  |
| Lower SPPB | 0.92 | 0.90 | 0.89 | 0.93 | 0.92 | 0.91 | 0.88 | 0.87 | 0.85 | | 0.89 | | 0.89 | | 0.89 | |  |
| Higher SPPB | 0.89 | 0.87 | 0.85 | 0.83 | 0.83 | 0.82 | 0.85 | 0.81 | 0.78 | | 0.90 | | 0.90 | | 0.91 | |  |
| **Number of WBs >10s** |  |  |  |  |  |  |  |  |  | |  | |  | |  | |  |
| Lower SPPB | 0.91 | 0.90 | 0.90 | 0.92 | 0.91 | 0.90 | 0.88 | 0.86 | 0.84 | | 0.82 | | 0.83 | | 0.83 | |  |
| Higher SPPB | 0.89 | 0.88 | 0.86 | 0.82 | 0.80 | 0.79 | 0.83 | 0.81 | 0.79 | | 0.86 | | 0.87 | | 0.88 | |  |
| **Number of WBs >30s** |  |  |  |  |  |  |  |  |  | |  | |  | |  | |  |
| Lower SPPB | 0.80 | 0.78 | 0.77 | 0.72 | 0.70 | 0.68 | 0.73 | 0.68 | 0.65 | | 0.69 | | 0.70 | | 0.71 | |  |
| Higher SPPB | 0.79 | 0.77 | 0.75 | 0.68 | 0.67 | 0.65 | 0.66 | 0.62 | 0.59 | | 0.61 | | 0.63 | | 0.65 | |  |
| **Number of WBs >60s** |  |  |  |  |  |  |  |  |  | |  | |  | |  | |  |
| Lower SPPB | 0.69 | 0.67 | 0.65 | 0.60 | 0.60 | 0.59 | 0.61 | 0.58 | 0.56 | | 0.67 | | 0.68 | | 0.68 | |  |
| Higher SPPB | 0.68 | 0.66 | 0.65 | 0.60 | 0.55 | 0.51 | 0.50 | 0.46 | 0.43 | | 0.50 | | 0.52 | | 0.54 | |  |
| **WB duration** |  |  |  |  |  |  |  |  |  | |  | |  | |  | |  |
| Lower SPPB | 0.82 | 0.81 | 0.79 | 0.81 | 0.80 | 0.80 | 0.80 | 0.79 | 0.78 | | 0.80 | | 0.80 | | 0.79 | |  |
| Higher SPPB | 0.86 | 0.86 | 0.85 | 0.76 | 0.74 | 0.73 | 0.80 | 0.78 | 0.76 | | 0.79 | | 0.80 | | 0.81 | |  |
| **P90 WB duration** |  |  |  |  |  |  |  |  |  | |  | |  | |  | |  |
| Lower SPPB | 0.74 | 0.73 | 0.72 | 0.82 | 0.81 | 0.80 | 0.70 | 0.70 | 0.69 | | 0.76 | | 0.77 | | 0.79 | |  |
| Higher SPPB | 0.77 | 0.77 | 0.76 | 0.67 | 0.62 | 0.58 | 0.62 | 0.56 | 0.51 | | 0.71 | | 0.73 | | 0.75 | |  |
| **WB duration bout to bout variability** |  |  |  |  |  |  |  |  |  | |  | |  | |  | |  |
| Lower SPPB | 0.73 | 0.73 | 0.72 | 0.65 | 0.65 | 0.66 | 0.68 | 0.65 | 0.62 | | 0.72 | | 0.74 | | 0.75 | |  |
| Higher SPPB | 0.77 | 0.75 | 0.74 | 0.71 | 0.69 | 0.67 | 0.61 | 0.58 | 0.55 | | 0.66 | | 0.68 | | 0.69 | |  |
| **Gait - Pace** | | | | | | | | | | | | | | | | | |
| **Walking speed in shorter (10-30s) WBs** |  |  |  |  |  |  |  |  |  |  | |  | |  | |  |  |
| Lower SPPB | 0.95 | 0.95 | 0.94 | 0.94 | 0.93 | 0.92 | 0.92 | 0.92 | 0.92 | | 0.91 | | 0.91 | | 0.92 | |  |
| Higher SPPB | 0.95 | 0.94 | 0.94 | 0.88 | 0.87 | 0.86 | 0.91 | 0.90 | 0.89 | | 0.95 | | 0.96 | | 0.96 | |  |
| **Walking speed in longer (>30s) WBs** |  |  |  |  |  |  |  |  |  |  | |  | |  | |  |  |
| Lower SPPB | 0.86 | 0.86 | 0.86 | 0.91 | 0.90 | 0.90 | 0.87 | 0.85 | 0.84 | | 0.83 | | 0.84 | | 0.85 | |  |
| Higher SPPB | 0.84 | 0.83 | 0.82 | 0.82 | 0.82 | 0.81 | 0.79 | 0.76 | 0.74 | | 0.91 | | 0.91 | | 0.90 | |  |
| **P90 walking speed in WBs >10 s** |  |  |  |  |  |  |  |  |  |  | |  | |  | |  |  |
| Lower SPPB | 0.90 | 0.90 | 0.91 | 0.95 | 0.95 | 0.94 | 0.92 | 0.91 | 0.90 | | 0.91 | | 0.90 | | 0.89 | |  |
| Higher SPPB | 0.88 | 0.88 | 0.88 | 0.90 | 0.90 | 0.89 | 0.91 | 0.89 | 0.88 | | 0.95 | | 0.95 | | 0.95 | |  |
| **P90 walking speed in longer (>30s) WBs** |  |  |  |  |  |  |  |  |  |  | |  | |  | |  |  |
| Lower SPPB | 0.87 | 0.87 | 0.87 | 0.91 | 0.91 | 0.91 | 0.88 | 0.87 | 0.85 | | 0.84 | | 0.84 | | 0.85 | |  |
| Higher SPPB | 0.86 | 0.85 | 0.84 | 0.87 | 0.87 | 0.86 | 0.83 | 0.80 | 0.78 | | 0.93 | | 0.92 | | 0.92 | |  |
| **Stride length in shorter (10-30s) WBs** |  |  |  |  |  |  |  |  |  |  | |  | |  | |  |  |
| Lower SPPB | 0.97 | 0.96 | 0.96 | 0.93 | 0.92 | 0.91 | 0.94 | 0.94 | 0.93 | | 0.92 | | 0.92 | | 0.93 | |  |
| Higher SPPB | 0.96 | 0.96 | 0.95 | 0.90 | 0.88 | 0.87 | 0.93 | 0.92 | 0.92 | | 0.95 | | 0.95 | | 0.96 | |  |
| **Stride length in longer (>30s)** **WBs** |  |  |  |  |  |  |  |  |  |  | |  | |  | |  |  |
| Lower SPPB | 0.89 | 0.89 | 0.89 | 0.89 | 0.88 | 0.86 | 0.90 | 0.89 | 0.87 | | 0.89 | | 0.89 | | 0.88 | |  |
| Higher SPPB | 0.85 | 0.84 | 0.84 | 0.82 | 0.80 | 0.78 | 0.82 | 0.80 | 0.78 | | 0.90 | | 0.90 | | 0.90 | |  |
| **Gait – Rhythm** | | | | | | | | | | | | | | | | | |
| **Cadence in all WBs** |  |  |  |  |  |  |  |  |  |  | |  | |  | |  |  |
| Lower SPPB | 0.96 | 0.96 | 0.96 | 0.97 | 0.97 | 0.96 | 0.95 | 0.95 | 0.95 | | 0.94 | | 0.94 | | 0.94 | |  |
| Higher SPPB | 0.96 | 0.96 | 0.96 | 0.95 | 0.95 | 0.94 | 0.95 | 0.94 | 0.93 | | 0.98 | | 0.98 | | 0.98 | |  |
| **Cadence in longer (>30s) WBs** |  |  |  |  |  |  |  |  |  |  | |  | |  | |  |  |
| Lower SPPB | 0.88 | 0.88 | 0.88 | 0.92 | 0.92 | 0.91 | 0.85 | 0.83 | 0.81 | | 0.86 | | 0.86 | | 0.86 | |  |
| Higher SPPB | 0.88 | 0.87 | 0.85 | 0.85 | 0.85 | 0.85 | 0.85 | 0.81 | 0.78 | | 0.92 | | 0.91 | | 0.91 | |  |
| **P90 cadence in longer (>30s) WBs** |  |  |  |  |  |  |  |  |  |  | |  | |  | |  |  |
| Lower SPPB | 0.88 | 0.88 | 0.88 | 0.91 | 0.91 | 0.91 | 0.84 | 0.82 | 0.80 | | 0.85 | | 0.84 | | 0.84 | |  |
| Higher SPPB | 0.86 | 0.85 | 0.84 | 0.87 | 0.86 | 0.85 | 0.85 | 0.81 | 0.77 | | 0.91 | | 0.91 | | 0.90 | |  |
| **Stride duration in all WBs** |  |  |  |  |  |  |  |  |  |  | |  | |  | |  |  |
| Lower SPPB | 0.92 | 0.92 | 0.91 | 0.88 | 0.87 | 0.86 | 0.87 | 0.86 | 0.86 | | 0.85 | | 0.87 | | 0.88 | |  |
| Higher SPPB | 0.93 | 0.93 | 0.92 | 0.85 | 0.83 | 0.82 | 0.87 | 0.84 | 0.81 | | 0.91 | | 0.91 | | 0.91 | |  |
| **Stride duration in longer (>30s) WBs** |  |  |  |  |  |  |  |  |  |  | |  | |  | |  |  |
| Lower SPPB | 0.85 | 0.85 | 0.85 | 0.85 | 0.84 | 0.82 | 0.81 | 0.80 | 0.78 | | 0.80 | | 0.81 | | 0.81 | |  |
| Higher SPPB | 0.86 | 0.85 | 0.84 | 0.80 | 0.78 | 0.76 | 0.82 | 0.79 | 0.75 | | 0.85 | | 0.85 | | 0.86 | |  |
| **Gait – Bout to bout variability** | | | | | | | | | | | | | | | | | |
| **Walking speed bout to bout variability between longer (>30s) WBs** |  |  |  |  |  |  |  |  |  |  | |  | |  | |  |  |
| Lower SPPB | 0.73 | 0.70 | 0.67 | 0.70 | 0.68 | 0.67 | 0.69 | 0.66 | 0.63 | | 0.53 | | 0.53 | | 0.54 | |  |
| Higher SPPB | 0.66 | 0.66 | 0.66 | 0.67 | 0.65 | 0.63 | 0.58 | 0.56 | 0.54 | | 0.65 | | 0.65 | | 0.66 | |  |
| **Stride length bout to bout variability between longer (>30s) WBs** |  |  |  |  |  |  |  |  |  |  | |  | |  | |  |  |
| Lower SPPB | 0.67 | 0.64 | 0.61 | 0.70 | 0.68 | 0.66 | 0.60 | 0.58 | 0.56 | | 0.71 | | 0.67 | | 0.64 | |  |
| Higher SPPB | 0.63 | 0.61 | 0.59 | 0.67 | 0.65 | 0.62 | 0.57 | 0.56 | 0.56 | | 0.59 | | 0.59 | | 0.60 | |  |
| **Cadence bout to bout variability** |  |  |  |  |  |  |  |  |  |  | |  | |  | |  |  |
| Lower SPPB | 0.87 | 0.87 | 0.87 | 0.88 | 0.87 | 0.87 | 0.88 | 0.88 | 0.88 | | 0.82 | | 0.81 | | 0.80 | |  |
| Higher SPPB | 0.88 | 0.87 | 0.86 | 0.84 | 0.84 | 0.84 | 0.83 | 0.83 | 0.82 | | 0.83 | | 0.84 | | 0.85 | |  |
| **Stride duration bout to bout variability** |  |  |  |  |  |  |  |  |  | |  | |  | |  | |  |
| Lower SPPB | 0.80 | 0.78 | 0.76 | 0.80 | 0.80 | 0.80 | 0.67 | 0.65 | 0.63 | | 0.71 | | 0.70 | | 0.69 | |  |
| Higher SPPB | 0.81 | 0.80 | 0.79 | 0.73 | 0.71 | 0.68 | 0.73 | 0.69 | 0.65 | 0.77 | | 0.78 | | 0.80 | |  |  |

COPD = chronic obstructive pulmonary disease; MS = multiple sclerosis; PD = Parkinson’s disease; PFF = proximal femoral fracture; WB = walking bout.
